# *Helicobacter pylori*-induced NAT10 stabilizes MDM2 mRNA via RNA acetylation to facilitate gastric cancer progression

**DOI:** 10.1186/s13046-022-02586-w

**Published:** 2023-01-06

**Authors:** Min Deng, Long Zhang, Wenying Zheng, Jiale Chen, Nan Du, Meiqi Li, Weiqing Chen, Yonghong Huang, Ning Zeng, Yuanbin Song, Yongming Chen

**Affiliations:** 1 Affiliated Cancer Hospital & Institute of Guangzhou Medical University, Guangzhou Key Laboratory of “Translational Medicine On Malignant Tumor Treatment”, Guangzhou, 510095 China; 2 Sun Yat-Sen University Cancer Center, State Key Laboratory of Oncology in South China, Collaborative Innovation Center for Cancer Medicine, Guangzhou, 510060 China; 3 First Department of Hepatobiliary Surgery, Zhujiang Hospital, Southern Medical University, Guangdong Provincial Clinical and Engineering Technology Center of Digital Medicine, Guangzhou, 510280 China

**Keywords:** Gastric cancer, N4-acetylcytidine, N-acetyltransferase 10, MDM2, p53, Helicobacter pylori

## Abstract

**Background:**

N4-acetylcytidine (ac4C), a widespread modification in human mRNAs that is catalyzed by the N-acetyltransferase 10 (NAT10) enzyme, plays an important role in promoting mRNA stability and translation. However, the biological functions and regulatory mechanisms of NAT10-mediated ac4C were poorly defined.

**Methods:**

ac4C mRNA modification status and NAT10 expression levels were analyzed in gastric cancer (GC) samples and compared with the corresponding normal tissues. The biological role of NAT10-mediated ac4C and its upstream and downstream regulatory mechanisms were determined in vitro and in vivo. The therapeutic potential of targeting NAT10 in GC was further explored.

**Results:**

Here, we demonstrated that both ac4C mRNA modification and its acetyltransferase NAT10 were increased in GC, and increased NAT10 expression was associated with disease progression and poor patient prognosis. Functionally, we found that NAT10 promoted cellular G2/M phase progression, proliferation and tumorigenicity of GC in an ac4C-depedent manner. Mechanistic analyses demonstrated that NAT10 mediated ac4C acetylation of MDM2 transcript and subsequently stabilized MDM2 mRNA, leading to its upregulation and p53 downregulation and thereby facilitating gastric carcinogenesis. In addition, *Helicobacter pylori* (*Hp*) infection contributed to NAT10 induction, causing MDM2 overexpression and subsequent p53 degradation. Further investigations revealed that targeting NAT10 with Remodelin showed anti-cancer activity in GC and augmented the anti-tumor activity of MDM2 inhibitors in p53 wild-type GC.

**Conclusions:**

These results suggest the critical role of NAT10-mediated ac4C modification in GC oncogenesis and reveal a previously unrecognized signaling cascade involving the Hp-NAT10-MDM2-p53 axis during GC development.

**Supplementary Information:**

The online version contains supplementary material available at 10.1186/s13046-022-02586-w.

## Background

Gastric cancer is one of the most common malignancies and accounts for the fourth leading cause of cancer-related death worldwide [1]. *Helicobacter pylori* infection, excessive intake of salt and nitrates, and other environmental risk agents contribute greatly to gastric cancer development [2]. In addition, genetic alterations and host factors are also implicated in GC initiation and progression [3]. Although surgical and adjuvant treatment approaches have improved, the prognosis of GC patients with advanced disease is still poor, with a 5-year survival rate of < 25% [4]. A better understanding of the underlying mechanisms of this lethal disease and the development of more effective targeted therapeutic strategies are necessary to improve the clinical outcomes of GC patients.

RNA undergoes distinct types of chemical modifications following its transcription, in a manner analogous to DNA and protein. Over 100 different chemical modifications have been identified in noncoding RNAs, and in recent years, some chemical modifications, such as N6-methyladenosine (m6A), 5-methylcytidine (m5C) [5, 6], N1-methyladenosine (m1A) [7], Am (2′-O-methyladenosine) [8] and N4-acetylcytidine (ac4C) [8, 9] have been reported to be widely present in mammalian mRNAs. Chemical modifications of mRNAs have been shown to contribute to gene expression control [10]. The best characterized mRNA modification is N6-methyladenosine (m6A). A large number of studies over the past few years have implicated this modification in a broad range of critical functions, including stem cell differentiation [11, 12], tissue development [13], sex determination [14] and tumor progression [15]. However, much less is known about other mRNA modifications, in particular, the ac4C mRNA modification. ac4C is the sole acetylation event that has been described to date in eukaryotic RNA. Its formation is catalyzed in humans by N-acetyltransferase 10 (NAT10), which is a singular enzyme with both acetyltransferase and RNA binding activities [16–18]. Recently, the mRNA modification ac4C was reported to promote mRNA stability and translation in human cells [9]. However, the status of ac4C modification and the underlying regulatory mechanisms in human diseases, including cancer, remain unknown.

In the present study, we investigated the status of mRNA modifications in GC and discovered that ac4C mRNA modification and its acetyltransferase NAT10 are significantly upregulated in GC. Further investigations revealed that NAT10 induced by *Hp* infection mediates ac4C acetylation of MDM2 mRNA and subsequently stabilizes MDM2 mRNA, causing consequent MDM2 upregulation and p53 degradation and thus facilitating gastric carcinogenesis. Our data also demonstrate that targeting NAT10 with Remodelin exhibits anti-gastric cancer activity and enhances the sensitivity of p53 wild-type GC to MDM2 inhibitors.

## Materials and methods

### Clinical sample collection

Clinical samples were collected from the Affiliated Cancer Hospital & Institute of Guangzhou Medical University. All specimens were obtained with informed consent and histopathologically confirmed by pathologists. Twenty pairs of fresh stomach adenocarcinoma tissues and adjacent normal mucosa tissues were used for HPLC–MS/MS, qRT–PCR assay and Western blot analyses, while 202 stomach adenocarcinoma tissues and 185 adjacent gastric tissues on tissue microarrays (TMAs) were used for IHC analysis. The clinical characteristics of the TMA samples are provided in Additional file 1: Table S1. The investigations involving human participants were approved by the Institutional Review Board of Affiliated Cancer Hospital & Institute of Guangzhou Medical University.

### CRISPR–Cas9-mediated knockout of NAT10

Disruption of the NAT10 gene in AGS cells was achieved using the CRISPR–Cas9 editing system. Briefly, the small guide RNA (sgRNA) targeting the genomic locus of NAT10 was synthesized and cloned into the lentiCRISPRv2 vector (52,961, Addgene). The recombinant vector was cotransfected with packaging plasmids into HEK293T packaging cells to generate lentivirus particles. AGS cells were infected with lentiviruses and selected in the presence of 2 mg/L puromycin (ant-pr-1, InvivoGen) for 1 week. Single cells were isolated using flow cytometry (BD FACS) and then seeded in 96-well plates. The knockout efficiency was determined in independent clones by targeted Sanger sequencing and Western blotting. The gRNA for NAT10 knockout was designed as follows: forward primer, 5-CACCG GGCTAGTGGTCATCCTCCTA-3; and reverse primer, 5-AAACTAGGAGGATGACCACTAGCC.

### Stable cell line generation

To establish cell lines stably re-expressing wild-type or mutant NAT10, NAT10-knockout AGS cells were transfected with the expression plasmids pNAT10, pK290A or pG641E using Lipofectamine 3000 (L3000015, Invitrogen) and were selected in the presence of 300 mg/L G418 (10,131,035, Gibco). To generate a cell line with stable MDM2 overexpression, NAT10 knockout AGS cells were transfected with the recombinant MDM2 plasmid or empty vector and incubated with 300 mg/L G418 for selection. BGC823 and MKN45 cells were infected with shNAT10 lentivirus, and stable NAT10-depleted cell lines were generated by selection with 2 mg/L puromycin.

### Mice xenograft experiments

All animal studies were approved by the Institutional Animal Care and Use Committee of Guangzhou Medical University. BALB/c nude mice (6–8 weeks old, male) were obtained from the Experimental Animal Center of Guangdong. To study the effect of NAT10 on tumor growth in vivo, control AGS cells, NAT10-knockout cells, or knockout cells expressing wild-type or mutant NAT10 (2 × 10^6^ cells per mouse) were subcutaneously implanted into nude mice. Tumor size was measured using the formula volume = (length × width^2^)/2. The mice were sacrificed, and tumors were removed and weighed at 25 days after inoculation. Furthermore, nude mice were intraperitoneally injected with the above cells to generate peritoneal dissemination. At 30 days after implantation, mice were sacrificed, and tumors in the abdominal cavity were assessed.

To study the in vivo therapeutic role of Remodelin, a NAT10 inhibitor, nude mice were subcutaneously injected with BGC823 cells (3 × 10^6^ cells per mouse). When tumors reached an average volume of 200 mm^3^, animals were intraperitoneally injected with different doses of Remodelin (S7641, Selleck) or vehicle control daily for 4 weeks. Mouse body weight was assessed every 5 days, and tumor size measurements were taken every 3 days with micro calipers. For survival experiments, the mice were followed for 120 days after the first drug treatment. To investigate the effect of combined treatment with HDM201 (S8606, Selleck) and Remodelin in vivo, BGC823 cells were subcutaneously implanted into nude mice. When tumors reached a volume of 150 to 250 mm^3^, animals were treated daily with vehicle control, HDM201 (10 mg/kg daily by oral gavage), Remodelin (30 mg/kg daily i.p.), or a combination for 4 weeks. Tumor size was monitored every 3 days as a surrogate for tumor burden. Changes in mouse body weight and survival time were measured.

### Statistical analysis

Data are presented as the means ± SD from three biological replicates. All statistical analysis was performed using GraphPad Prism 8 and SPSS 17 software. The significance of the differences between groups was determined by two-tailed t-test. The Pearson correlation test was used to calculate the correlations between the two groups. The χ2 test was performed to evaluate the statistical significance of differences in IHC staining of human GC and normal tissues. The survival curves were measured using the Kaplan–Meier method, and the hazard ratio for survival was estimated by Cox regression analysis. Values of *P* < 0.05 were considered statistically significant.

## Results

### High NAT10 expression in gastric cancer is correlated with increased mRNA acetylation and poor patient prognosis

**Fig. 1:**
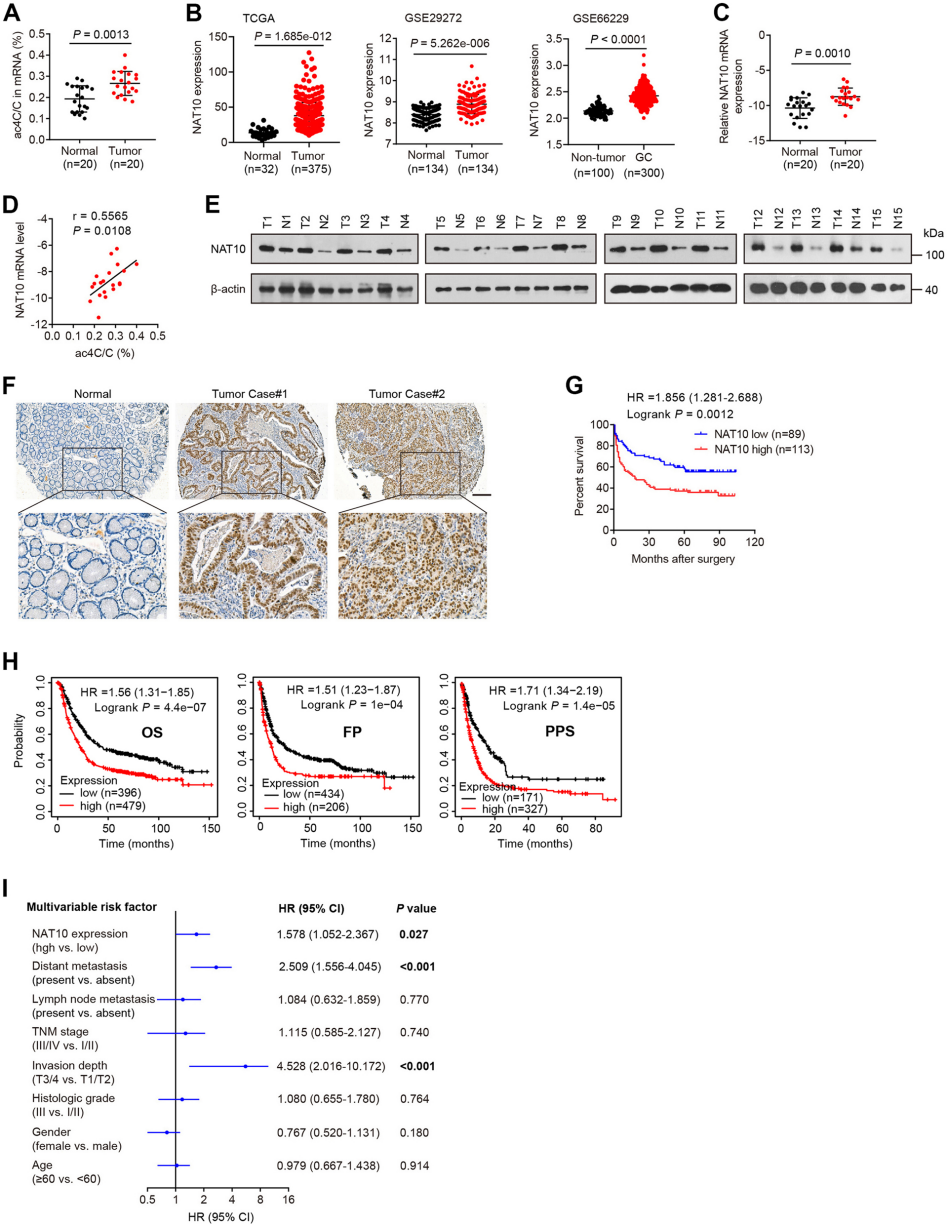
ac4C mRNA acetylation and NAT10 expression are upregulated in gastric cancer. **A** The ac4C/C ratio in polyA( +) mRNA isolated from 20 GCs (tumor) and paired normal gastric mucosal tissues (Normal) was quantified by HPLC–MS/MS. **B** The expression levels of NAT10 in GC vs. nontumor gastric tissues from the TCGA data and GEO datasets. **C** qRT–PCR analysis of NAT10 mRNA levels in 20 pairs of GC and adjacent normal tissues. **A**-**C** *P* values were calculated using a two-tailed t-test. **D** Pearson correlation analysis showing that the ac4C acetylation level is correlated with the mRNA expression of NAT10 in GC specimens. **E** Western blot assay of NAT10 protein levels in 15 GC tissues and paired normal tissues. T, gastric tumor; N, adjacent normal tissue. **F** IHC analyses of NAT10 expression in 202 GC and 185 adjacent normal tissues on tissue microarrays. Scale bar: 150 μm. **G** Overall survival curves for GC patients in the TMA cohort with high or low expression of NAT10. **H** Kaplan–Meier analyses of OS, FP and PPS for GC patients based on NAT10 expression using the online tool Kaplan–Meier Plotter. **I** Multivariate Cox analysis of factors associated with OS in TMA samples. CI, confidence interval; HR, hazard rate. Error bars, SD

To explore the functional roles of mRNA modifications in GC, we first examined 9 nucleoside modifications in mRNA from 20 paired GC and adjacent normal gastric mucosa tissues via HPLC–MS/MS assay. We found a significant increase in ac4C on mRNA in gastric tumor tissues compared with that in adjacent normal tissues (Fig. 1A and Additional file 1: Fig. S1A). Based on the fact that the ac4C modification is catalyzed by NAT10 [16, 17], we sought to determine whether the elevated ac4C mRNA levels in GC are caused by altered expression of NAT10. Upon analysis of the TCGA data and GEO datasets, we observed that NAT10 was significantly upregulated in gastric and other tumor tissues relative to the corresponding normal tissues (Fig. 1B and Additional file 1: Fig. S1B). We then performed quantitative RT–PCR (qRT–PCR) analyses for 20 matched pairs of GCs and adjacent normal tissues and observed upregulation of NAT10 in GCs compared to the corresponding normal tissues (Fig. 1C). Moreover, increased NAT10 expression correlated with elevated ac4C levels in GC tissues (Fig. 1D). Subsequent Western blot analysis validated the upregulation of NAT10 in GC tissues and cell lines at the protein level (Fig. 1E and Additional file 1: Fig. S2A). Immunohistochemistry (IHC) of GC tissue microarrays further validated the elevated NAT10 expression in GC samples (Fig. 1F and Additional file 1: Fig. S1C). More importantly, high NAT10 expression was significantly associated with tumor grade, invasion depth, clinical stage and metastasis as well as with inferior overall survival (OS) of GC patients (Fig. 1G and Additional file 1: Table S2). Bioinformatics analysis of the GEO data using the Kaplan–Meier Plotter further demonstrated that high expression of NAT10 was correlated with shorter overall survival, first progression (FP) and post progression survival (PPS) in gastric cancer patients (Fig. 1H). In addition, NAT10 was found to be an independent risk factor for overall survival by multivariate Cox analysis (Fig. 1I). These data suggest that enhanced mRNA acetylation and NAT10 overexpression may promote the development and progression of GC.

### NAT10 promotes gastric cancer cell proliferation and growth in an ac4C-dependent manner

**Fig. 2:**
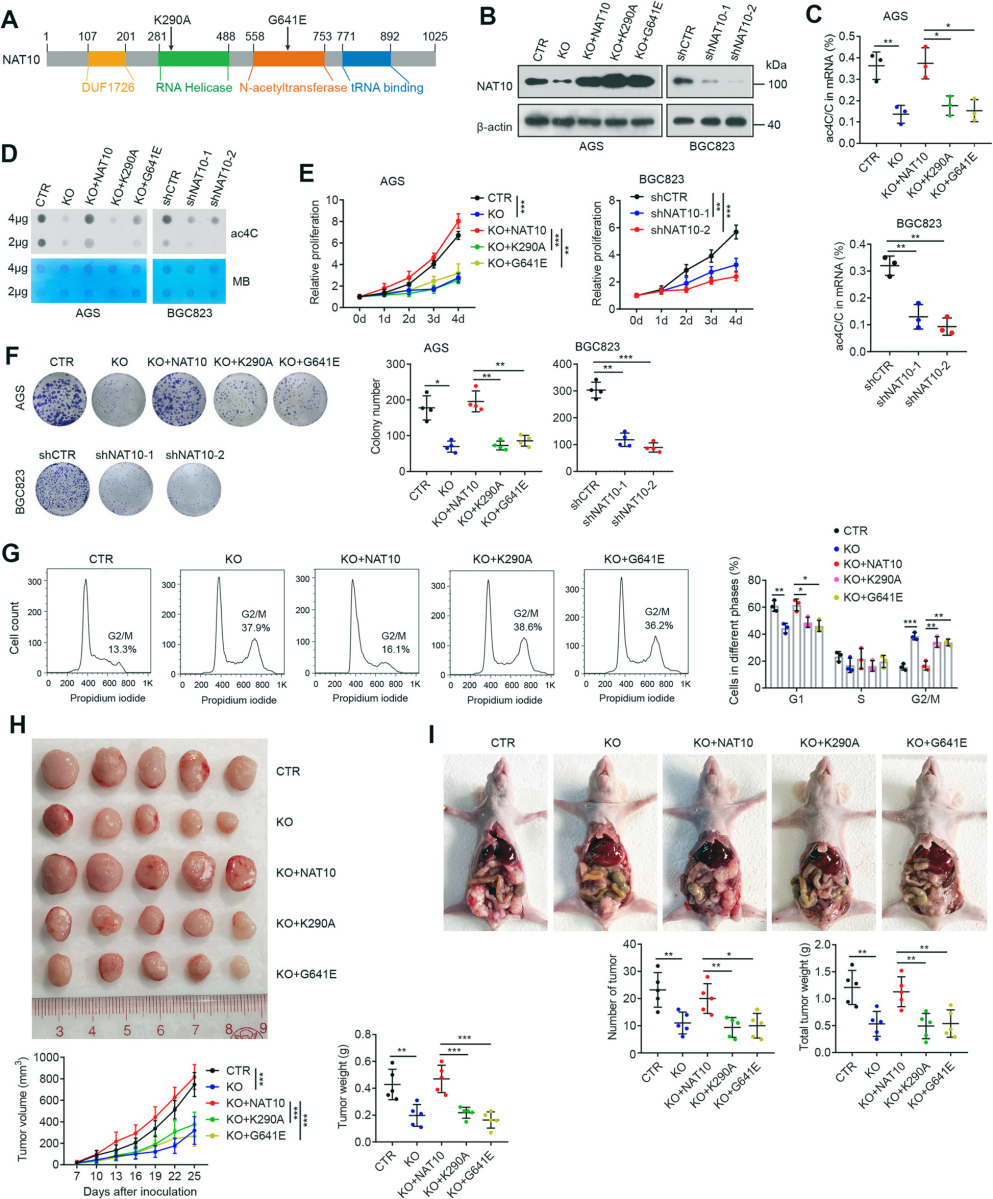
NAT10 depletion inhibits GC cell proliferation and in vivo tumor growth. **A** Schematic of NAT10 with its known domains. Arrows indicate point mutations. **B** Western blot analysis was performed to confirm the level of NAT10 in control AGS cells, NAT10-knockout cells, knockout cells rescued with stable expression of wild-type or mutant NAT10, and BGC823 cells stably expressing NAT10 shRNAs or control shRNA. **C**-**F** The ac4C mRNA levels were tested by HPLC–MS/MS (**C**) and ac4C dot blot (**D**) analyses, and proliferation capacities were detected by CCK-8 (**E**) and colony formation (**F**) assays in the above cells. **G** The cell cycle distribution was assessed in the indicated cells by flow cytometry. **H** and **I** NAT10 knockout inhibited subcutaneous tumor growth (**H**) and the formation of tumor nodules in the peritoneal cavity (**I**), while overexpression of wild-type NAT10, but not of the K290A or G641E mutants, offset these effects (*n* = 5 mice/group). CTR, control; KO, NAT10 knockout; shCTR, control shRNA; shNAT10, NAT10 shRNA. Error bars indicate the SD. *P < 0.05, **P < 0.01, *** *P* < 0.001 (two-tailed t-test)

To investigate the roles of NAT10-mediated ac4C modification on gastric carcinogenesis, we used the CRISPR/Cas9 technique to establish NAT10-knockout AGS cell lines (Fig. 2B and Additional file 1: Fig. S2B). As expected, NAT10 knockout dramatically reduced global ac4C modification in both total RNA and mRNA (Fig. 2C, D and Additional file 1: Fig. S2C-E). Importantly, NAT10 knockout decreased cell proliferation, colonic growth and invasion and induced substantial G2/M arrest and moderate apoptosis (Fig. 2E-G and Additional file 1: Fig. S3D, E, F, H). Similar phenomena were observed when NAT10 was knocked down by lentiviral-mediated shRNA in BGC823 and MKN45 cells (Fig. 2B-F and Additional file 1: Fig. S3). To test whether NAT10-promoted malignant phenotypes of cancer cells were dependent on its ac4C activity, NAT10-knockout cells were used to express wild-type NAT10 or mutant NAT10 lacking a functional acetyltransferase domain (G641E) or RNA helicase domain (K290A) (Fig. 2A, B) due to point mutations (K290A or G641E) previously shown to disrupt the RNA acetyltransferase function of NAT10 [19–21]. As shown in Fig. 2C-G and Additional file 1: Fig. S2C, S3E-F, re-expression of wild-type NAT10 in NAT10-knockout cells effectively rescued ac4C acetylation and corresponding changes to cancer cell physiology, whereas neither of the two NAT10 mutants exhibited these effects, suggesting that the RNA ac4C modification function of NAT10 is indispensable for its role in promoting gastric carcinogenesis. To further confirm this, Flag-tagged NAT10 Δhelicase lacking the RNA helicase domain was transiently transfected into NAT10-knockout cells. The results showed that the expression of NAT10 lacking the helicase domain failed to effectively reconstitute either RNA ac4C or cell proliferation (Additional file 1: Fig. S2D-E, S3D).

To evaluate the oncogenic role of NAT10 in gastric cancer in vivo, we applied both a subcutaneous xenograft model and a peritoneal dissemination model. In the subcutaneous xenograft model, NAT10 knockout significantly reduced the proliferation and growth of xenograft tumors, which could be offset by re-expression of wild-type NAT10 but not its mutant forms (Fig. 2H and Additional file 1: Fig. S4). The peritoneal dissemination assay showed that NAT10 knockout significantly reduced the formation of tumor nodules in the peritoneal cavity, and this effect could be reversed by the overexpression of wild-type NAT10 but not its mutant counterparts (Fig. 2I). Collectively, these results demonstrate the ac4C-dependent oncogenic role of NAT10 in GC progression.

### NAT10-mediated ac4C modification maintains the stability of the MDM2 transcript

**Fig. 3:**
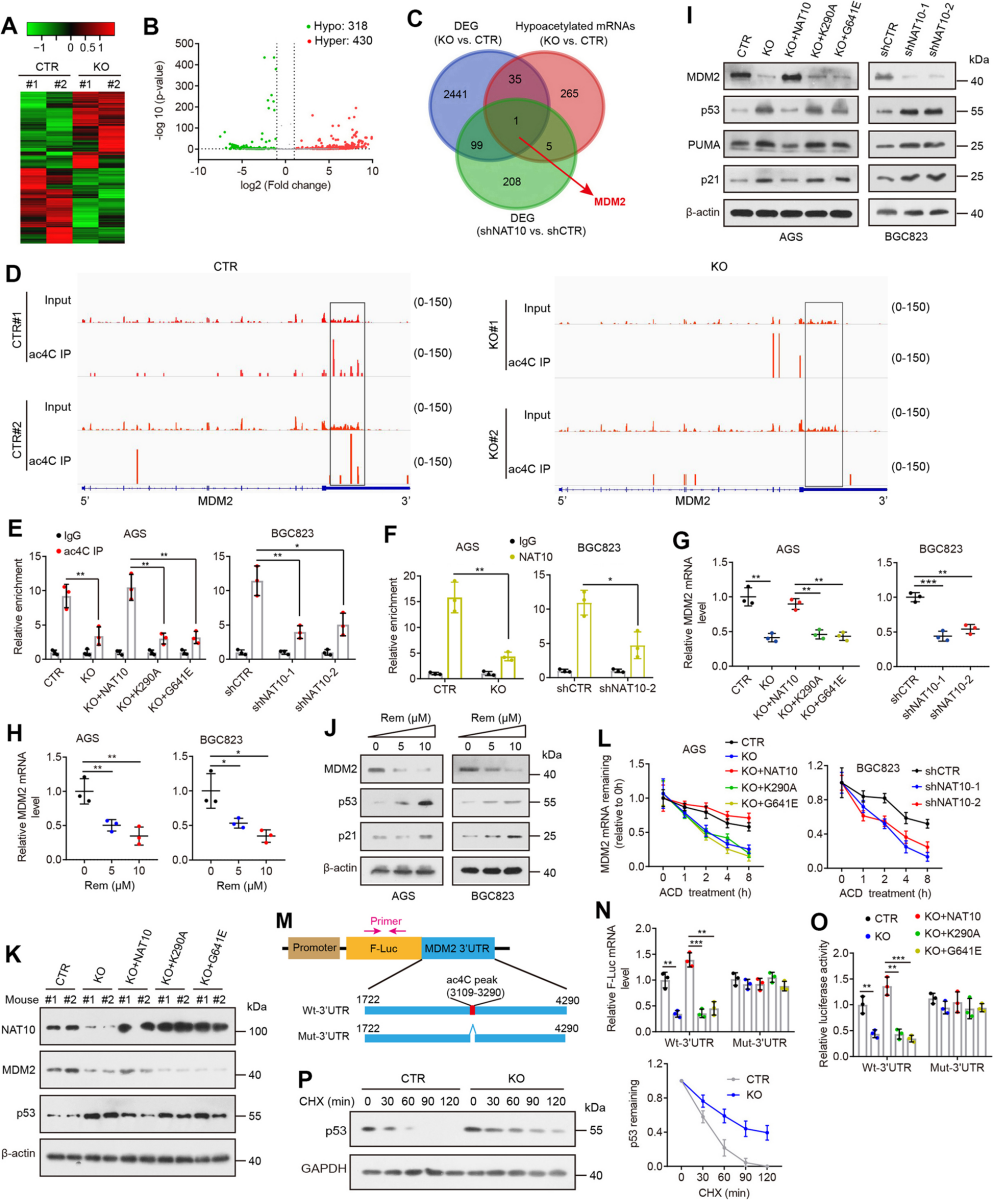
NAT10 maintains the stability of MDM2 mRNA via ac4C modification. **A** Heat map showing differentially expressed genes identified by RNA-seq in NAT10-knockout cells relative to the control cells. Green and red indicate low and high mRNA levels, respectively. **B** Volcano plot of altered ac4C peaks within mRNA transcripts between NAT10-knockout and control cells. **C** A Venn diagram shows overlapping mRNA transcripts that were both differentially expressed (DEG) and hypoacetylated upon NAT10 knockout and differentially expressed genes (DEG) following NAT10 knockdown. **D** The ac4C peak was enriched in the 3′UTR of MDM2 from the acRIP-seq data. Squares indicate a significant decrease in the ac4C peak in NAT10-knockout cells relative to control AGS cells. **E** The relative ac4C levels of the MDM2 transcript were evaluated in the indicated cells by acRIP-qPCR. **F** RIP assay with anti-NAT10 and anti-IgG antibodies was carried out to analyze the relative NAT10 enrichment in MDM2 mRNA. **G** The mRNA levels of MDM2 in the indicated cells. **H** MDM2 mRNA levels were measured in cells treated with Remodelin for 24 h. **I** and **J** MDM2, p53, p21 and PUMA were detected by Western blotting in the indicated cells. **K** Western blot analysis of NAT10, MDM2 and p53 in xenograft tumor tissues. **L** MDM2 mRNA stability assessment in the indicated cells treated with 5 μg/mL actinomycin D (ACD). **M** A schematic diagram illustrating the luciferase reporter plasmids containing the wild-type (Wt) MDM2 3’UTR fragment or its mutant (Mut) counterpart that lacks the ac4C peak region. **N** and **O** The relative mRNA expression (**N**) and activity (**O**) of firefly luciferase fused with the wild-type or mutant MDM2 3′UTR in control AGS cells, NAT10-knockout cells and knockout cells re-expressing wild-type or mutant NAT10. **P** Immunoblot of p53 protein and quantification of the relative level of p53 at the indicated time in control and NAT10-knockout AGS cells after treatment with 100 μg/ml cycloheximide (CHX) to block protein synthesis. Error bars represent the SD from three independent experiments. *P < 0.05, **P < 0.01, *** *P* < 0.001. ns, not significant. All *P* values were determined by two-tailed t-test

To explore the molecular mechanism by which NAT10 promotes GC progression, we performed RNA sequencing (RNA-seq) and ac4C-RNA immunoprecipitation sequencing (acRIP-seq) assays in stable NAT10 knockout and control AGS cells with independent biological replicates. RNA-seq revealed 2576 differentially expressed genes upon NAT10 knockout (Fig. 3A), which were found to be significantly enriched in gene sets involved in cell mitotic division, DNA replication, cell cycle, cell proliferation and so on by Gene Ontology (GO) enrichment analysis (Additional file 1: Fig. S5A). Notably, KEGG pathway analysis revealed that the cell cycle, p53 signaling pathway and DNA replication were affected by NAT10 knockout (Additional file 1: Fig. S5B). In acRIP-seq analysis, we identified on average 4,342 ac4C peaks in 3,591 transcripts and 2,709 ac4C peaks in 2,348 transcripts in control and NAT10-KO cells, respectively (Additional file 1: Fig. S5C). ac4C peaks were enriched in the 3′UTRs and coding sequences (Additional file 1: Fig. S5D, E). Among these peaks, 318 peaks within 306 mRNAs displayed hypoacetylation, and 430 peaks within 413 mRNAs showed hyperacetylation in NAT10-knockout cells relative to those in control cells (Fig. 3B and Additional file 2: Table S3). Since NAT10 positively mediates ac4C modification, only ac4C peaks with decreased abundance (termed ac4C hypo-peaks) upon NAT10 knockout were theoretically anticipated to include genuine targets of NAT10. We assessed whether these ac4C hypo-peaks were associated with differentially expressed mRNA genes in the RNA-seq analysis. The intersection of 318 hypo-peaks (within 306 mRNAs) with the 2576 differentially expressed genes identified by RNA-seq led to the identification of 36 candidate genes (Fig. 3C). To narrow down the scope of downstream targets, we additionally performed an RNA-seq assay in BGC823 cells with or without NAT10 knockdown and found that 313 coding genes were differentially expressed following NAT10 knockdown (Additional file 1: Fig. S5F), among which only one gene, MDM2, displayed ac4C hypoacetylation upon NAT10 knockout and consistent mRNA downregulation following either NAT10 knockout or knockdown (Fig. 3C).

**Fig. 4:**
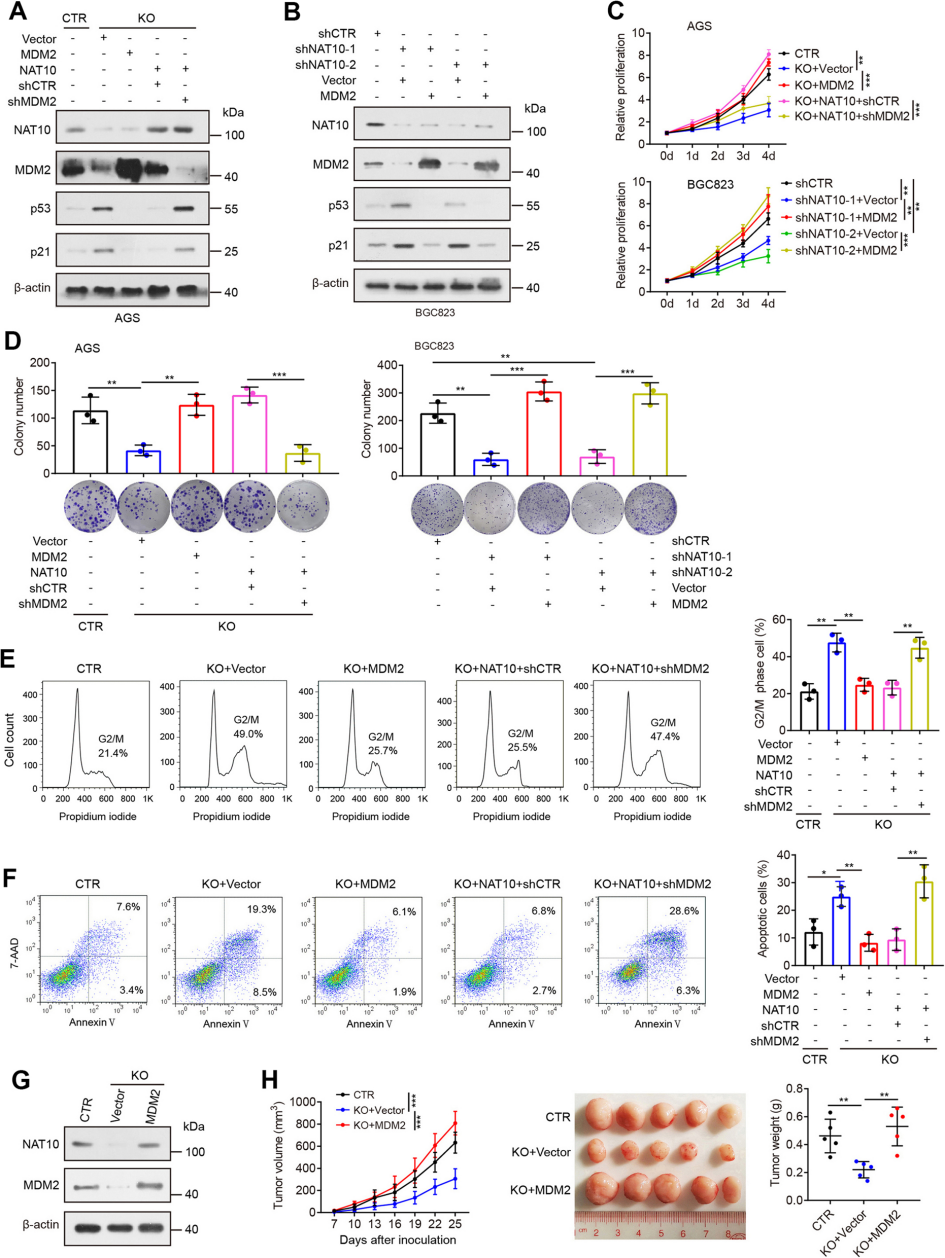
MDM2 is a major contributor to the function of NAT10 in gastric carcinogenesis. **A** Overexpression of MDM2 inhibited the upregulation of p53 and p21 proteins in NAT10-knockout AGS cells, while knockdown of MDM2 effectively reversed the inhibitory effect of NAT10 overexpression on p53 and p21. **B** MDM2 overexpression reversed the upregulation of p53 and p21 proteins by NAT10 knockdown in BGC823 cells. **C** and **D** The effects of NAT10 depletion on cell proliferation (**C**) and colonic growth (**D**) were rescued by transfection with MDM2, whereas cell proliferation and colonic growth of NAT10-overexpressing cells were prevented by knockdown of MDM2. **E** and **F** Cell cycle (**E**) and apoptosis (**F**) were measured in the indicated cells by flow cytometry. **G** MDM2 and NAT10 proteins were evaluated in NAT10-knockout AGS cells stably expressing MDM2 or vector control. **H** MDM2 overexpression rescued the impaired capacity of tumor growth triggered by NAT10 knockout (*n* = 5 mice/group). Error bars, SD. *P < 0.05, **P < 0.01, ***P < 0.001 using a two-tailed t-test

Our ac4C-seq data revealed that MDM2 mRNA was modified by ac4C in its 3′UTR and that NAT10 knockout caused a significant decrease in ac4C levels (Fig. 3D). To confirm this, we applied acRIP-qPCR to examine the effects of NAT10 expression changes on ac4C levels in MDM2 mRNA. In accordance with the acRIP-seq results, significant ac4C enrichment of MDM2 mRNA was observed, and importantly, ac4C levels of MDM2 mRNA were significantly decreased in NAT10-depleted cells, while this effect was reversed by re-expression of wild-type NAT10 but not its mutants (Fig. 3E and Additional file 1: Fig. S6A). Similar to genomic NAT10 deletion, pharmacologic inhibition of NAT10 with Remodelin, a recently described NAT10 inhibitor [19], reduced global ac4C levels in both total RNA and mRNA and, more importantly, suppressed MDM2 ac4C modification (Additional file 1: Fig. S6B-D). In addition, RNA immunoprecipitation (RIP) followed by qRT–PCR assays showed that NAT10 could be significantly enriched in MDM2 mRNA (Fig. 3F). We subsequently measured the change in the mRNA and protein levels of MDM2 upon NAT10 knockout or knockdown. Consistent with the RNA-seq data, knockout of NAT10 substantially reduced MDM2 expression at both the mRNA and protein levels, whereas overexpression of wild-type NAT10, but not the mutants K290A or G641E, could enhance MDM2 expression (Fig. 3G, I), indicating that NAT10 mainly affects MDM2 expression through its RNA acetylation activity. Decreased MDM2 was also observed when we knocked down NAT10 in BGC823 and MKN45 cells (Fig. 3G, I and Additional file 1: Fig. S6E, F). Not surprisingly, treatment with Remodelin resulted in a significant reduction in MDM2 expression levels in a dose-dependent manner (Fig. 3H, J). Conversely, NAT10 expression was not altered by MDM2 overexpression or knockdown (Fig. 4A, B, G). Similar to the data obtained from human cell lines, the NAT10-knockout tumors showed a reduction in ac4C modification of the MDM2 transcript and decreased MDM2 expression compared to those in the control tumors, whereas the tumors with re-expression of wild-type NAT10 exhibited enhanced levels of MDM2 mRNA acetylation and MDM2 expression (Fig. 3K and Additional file 1: Fig. S6G, H). These data strongly support MDM2 as a bona fide target of NAT10.

Considering the role of NAT10 in regulating MDM2 mRNA levels, we evaluated whether NAT10 may affect MDM2 transcription and mRNA export. A luciferase reporter assay showed that NAT10 ablation did not affect MDM2 promoter activity (Additional file 1: Fig. S6I), suggesting that transcription of MDM2 was not affected by ac4C. Furthermore, no obvious change in the subcellular localization of MDM2 mRNA between the control and NAT10-KO cells was found (Additional file 1: Fig. S6J). Since transcription and mRNA export cannot explain the reduced MDM2 expression in NAT10-depleted cells, we then asked whether NAT10 influences MDM2 mRNA stability. In fact, depletion of NAT10 resulted in a noticeable decrease in the stability of the MDM2 transcript, while overexpression of wild-type NAT10 but not its mutants offset this effect (Fig. 3L). We also observed an obvious decrease in MDM2 mRNA stability in AGS and BGC823 cells with Remodelin treatment (Additional file 1: Fig. S6K). To study the potential roles of ac4C acetylation on the MDM2 3′UTR, we generated luciferase reporters containing a firefly luciferase, followed by the wild-type (Wt) MDM2 3′UTR or its mutant (Mut) counterpart. For the Mut reporters, the region covered by the ac4C peak (181 bases in length, putative ac4C sites) was deleted to eliminate the effect of ac4C acetylation (Fig. 3M). We found that depletion of NAT10 caused a markable reduction in both mRNA expression and activity of firefly luciferase when fused with the wild-type MDM2 3′UTR, and this effect was offset when firefly luciferase was fused with the mutant MDM2 3′UTR (Fig. 3N, O). Furthermore, overexpression of wild-type NAT10 rather than its mutant counterparts substantially increased luciferase mRNA levels and its activity of the reporter construct containing the wild-type MDM2 3′UTR, while the lack of the ac4C peak region in the MDM2 3′UTR abrogated this effect (Fig. 3N, O). In summary, our results indicate the regulation of MDM2 is under the control of NAT10-guided ac4C modifications.

As MDM2, an E3 ubiquitin ligase, targets p53 for proteasomal degradation and functions as a negative regulator of p53 [22], we tested whether NAT10 affects the p53 pathway in the p53 wild-type cell lines AGS and BGC823. As expected, p53 protein levels were significantly induced following genomic deletion or chemical inhibition of NAT10 but were decreased after re-expression of wild-type NAT10 but not its mutant forms (Fig. 3I, J). Similar results were observed in the xenograft tumor tissues (Fig. 3K). Even though p53 protein levels were changed upon regulation of NAT10 expression, the p53 (TP53) mRNA levels remained unchanged (Additional file 1: Fig. S6L), demonstrating that NAT10 reduces the expression of p53 at the protein but not the mRNA. Consistent with these findings, both mRNA and protein levels of the p53 target genes, p21 (CDKN1A) and PUMA, were also increased after depletion or inhibition of NAT10 and reduced after re-expression of NAT10 (Fig. 3I, J and Additional file 1: Fig. S6L, M). NAT10 was reported to acetylate p53 protein at K120 in CRC cells in a previous study [23]. In contrast, we and another group [9] observed that NAT10 failed to affect p53 acetylation (Additional file 1: Fig. S6N), which may be due to the cell type-specific role of NAT10 or bias relating to the experimental conditions. We examined whether NAT10 ablation affects p53 protein degradation and found that NAT10 knockout significantly increased the stability of endogenous p53 protein (Fig. 3P), indicating that NAT10 destabilizes p53 protein.

### MDM2 mediates NAT10-induced malignant cell phenotypes

We further explored the biological significance of MDM2 in the tumor-promoting function of NAT10. Ectopic expression of MDM2 significantly reduced the induction of p53 and its target p21 elicited by NAT10 depletion, whereas knockdown of MDM2 effectively abolished the inhibitory effect of NAT10 overexpression on the p53 pathway (Fig. 4A, B), indicating that NAT10 can destabilize p53 protein via MDM2. We subsequently examined whether MDM2 affects NAT10-mediated malignant cell phenotypes. Indeed, overexpression of MDM2 significantly rescued the hypoproliferative phenotype of NAT10-deficient GC cells. In contrast, MDM2 silencing largely abrogated the effect of NAT10 overexpression on cell growth/proliferation (Fig. 4C, D). Moreover, MDM2 overexpression significantly abolished G2/M arrest and apoptosis mediated by NAT10 depletion. In contrast, MDM2 silencing resulted in an induction of cell cycle arrest and apoptosis in NAT10-overexpressing cells (Fig. 4E, F). Thereafter, further in vivo rescue experiments were conducted, and our data demonstrated that the impaired potential of in vivo tumor growth triggered by NAT10 knockout could be restored by stable MDM2 overexpression (Fig. 4G, H), showing that MDM2 mediates the oncogenic functions of NAT10.

**Fig. 5:**
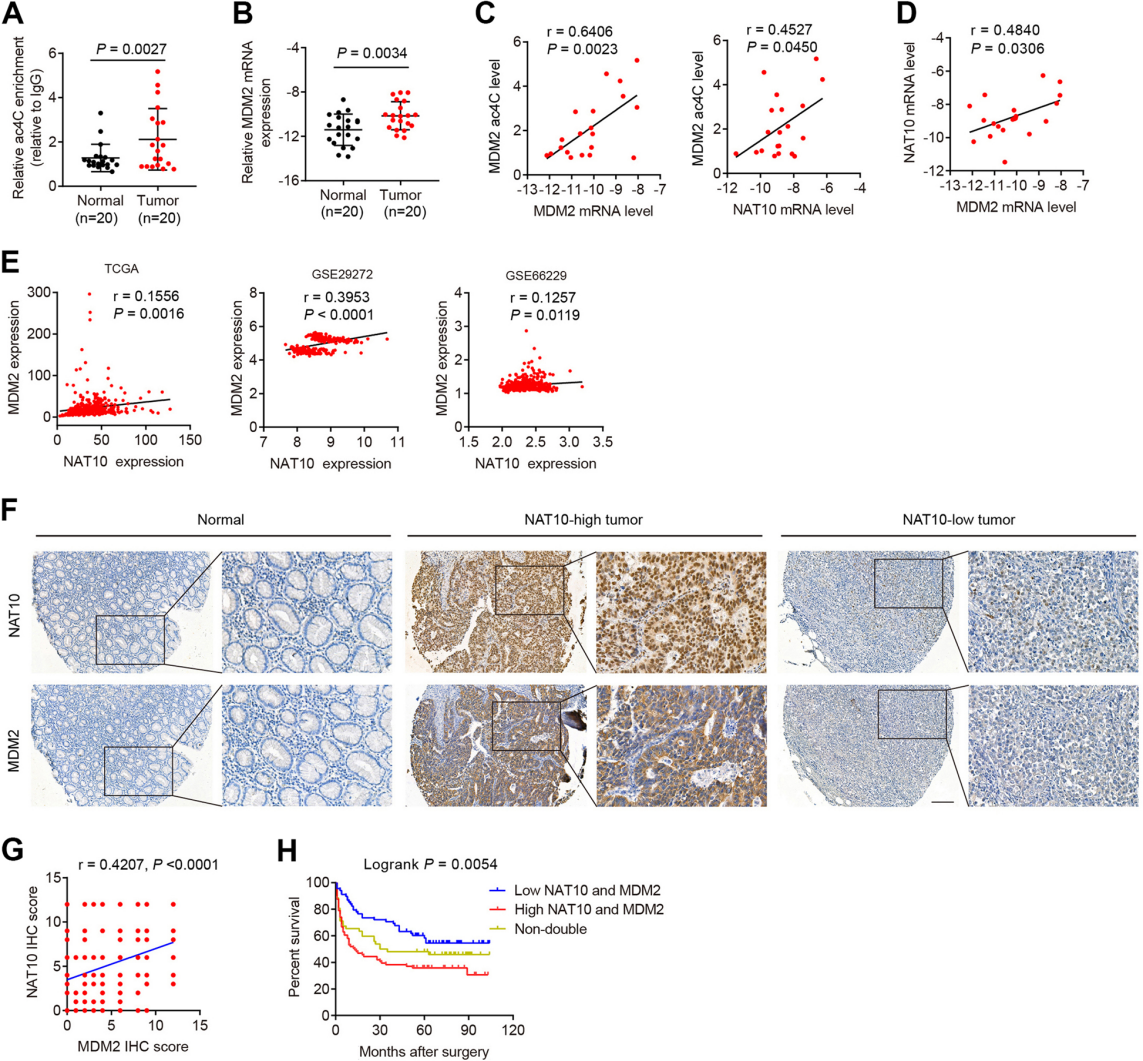
NAT10 overexpression correlates with high levels of MDM2 ac4C modification and MDM2 expression in gastric cancer specimens. **A** and **B** The ac4C levels of the MDM2 transcript were measured by acRIP-qPCR analysis (**A**), and MDM2 mRNA levels were tested by qRT–PCR (**B**) in 20 GC and paired normal gastric mucosal tissues. The differences were determined with a two-tailed t-test. **C** ac4C levels of MDM2 mRNA were positively correlated with MDM2 and NAT10 expression in GC specimens. **D** The graph shows a significant correlation of NAT10 mRNA with MDM2 expression in GCs. **E** The TCGA and GEO datasets shows that NAT10 and MDM2 levels were correlated in GC tissues. r and *P* values were determined by Pearson correlation test (**C**-**E**). **F**–**H** Representative images of IHC staining of NAT10 and MDM2 in normal gastric tissues and two GC samples with high or low expression of both proteins are shown (**F**). Scale bar, 150 μm. NAT10 expression was positively correlated with MDM2 expression (**G**). r and *P* values were calculated using the Pearson correlation test. Kaplan–Meier analysis indicates the correlation between the combination of high expression of NAT10 and MDM2 and poorer OS (**H**). Error bars, SD

We next sought to verify whether our findings could be extended to gastric cancer patients. The results showed that GC specimens displayed enhanced levels of both MDM2 ac4C and MDM2 mRNA expression compared to those in paired normal gastric specimens (Fig. 5A, B). Further correlative analysis of the ac4C modification level within MDM2 showed a significant association with MDM2 mRNA levels as well as with NAT10 expression in GC tissues (Fig. 5C), further supporting the notion that NAT10-mediated ac4C modification stabilizes MDM2 mRNA. Moreover, NAT10 mRNA expression positively was associated with MDM2 mRNA expression (Fig. 5D). This correlation was true in gastric cancer sample cohorts based on analysis of the mRNA expression data from the TCGA and GEO datasets (Fig. 5E). IHC staining of MDM2 in the TMA cohort demonstrated high expression of MDM2 in 102 of 202 (50.5%) GC tissues (Fig. 5F and Additional file 1: Fig. S1D). More importantly, MDM2 protein expression was positively associated with NAT10 protein in GC tissues (Fig. 5F). In addition, Kaplan–Meier survival curves showed that high expression of both NAT10 and MDM2 predicted the poorest overall survival (Fig. 5H). These data further support our findings that the NAT10/MDM2 axis promotes GC progression and leads to worsened overall survival.

### *Hp* infection induces NAT10 expression and regulates p53 stability

**Fig. 6:**
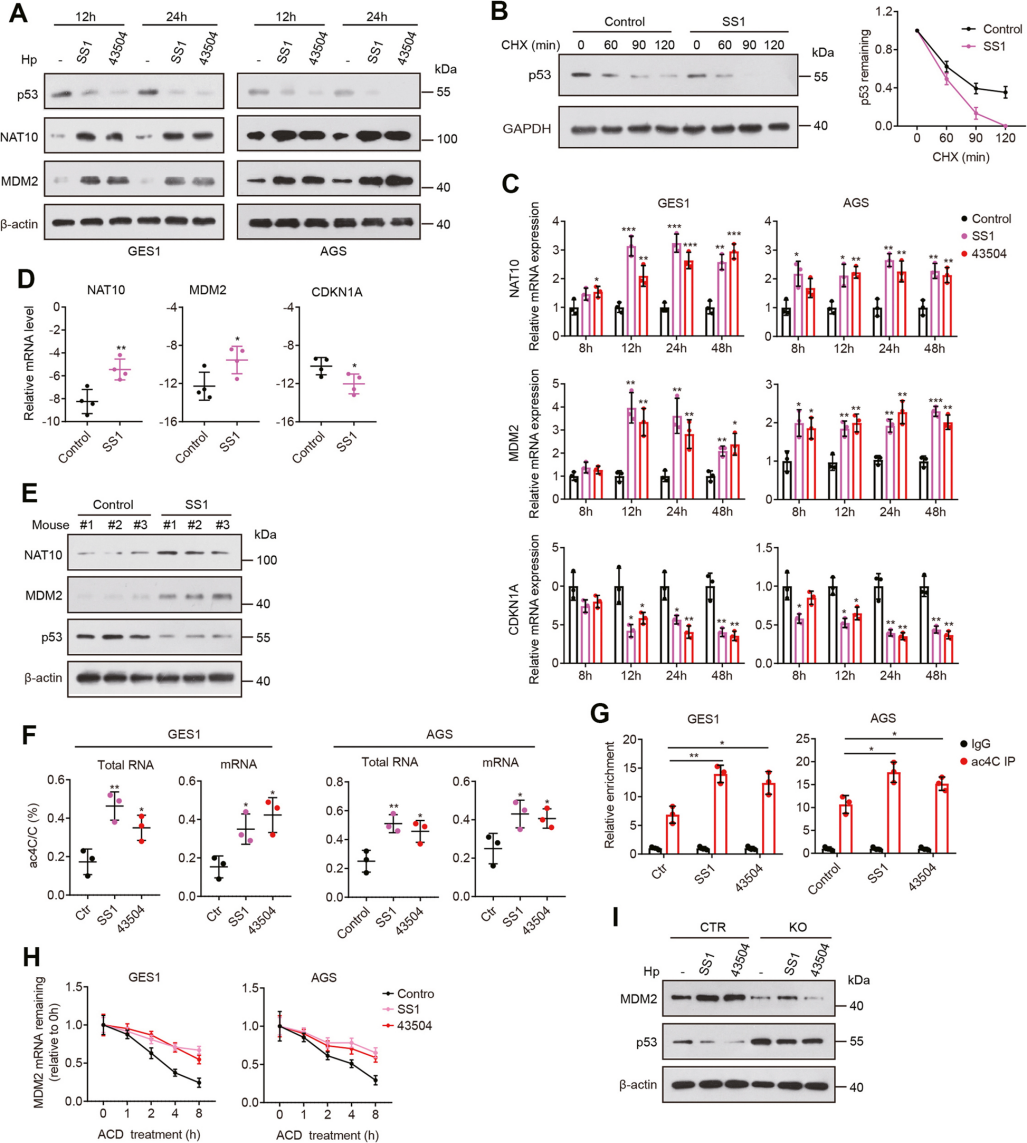
*Hp* infection enhances NAT10 expression and regulates p53 stability. **A** Western blot analysis of p53, NAT10 and MDM2 following coculture of GES1 and AGS cells with *Hp*. **B** The stability of p53 protein was determined in GES1 cells cocultured with *Hp* SS1 using the cycloheximide chase method. **C** qRT–PCR was performed to analyze NAT10, MDM2 and CDKN1A expression in the indicated cells. **D** and **E** qRT–PCR analysis of NAT10, MDM2 and CDKN1A expression (**D**) and Western blot analysis of NAT10, MDM2 and p53 (**E**) in gastric tissues from mice challenged with *Hp* SS1 or Brucella broth (Control) for 3 weeks. **F** The global ac4C acetylation levels in mRNA and total RNA from GES1 and AGS cells cultured in the presence or absence of *Hp*. **G** The ac4C levels of MDM2 mRNA in cells treated as described in H. **H** The stability of MDM2 mRNA was assessed in the indicated cells treated with 5 μg/mL ACD. The MDM2 mRNA abundance relative to that of GAPDH as quantified by qRT–PCR. **I** Protein levels were tested in NAT10-knockout and control cells following coculture with *Hp*. SS1, *Hp* strain SS1; 43,504, *Hp* strain ATCC43504. Error bars, SD. *P < 0.05, **P < 0.01, ***P < 0.001 (two-tailed t-test)

*Hp* is a major risk factor for gastric cancer [24–26]. Previous studies have shown that *Hp* infection promotes proteasomal degradation of p53 in gastric epithelial cells, which results in downregulation of p53 protein [27–29]. Given that our results suggest that NAT10 maintains the stability of MDM2 mRNA via ac4C modification and consequently destabilizes the p53 protein, we asked whether *Hp* infection can affect NAT10 expression to regulate p53 stability. To answer this question, we first measured p53 protein in GES1 normal gastric epithelial cells and AGS gastric cancer cells cocultured with two *Hp* standard strains, SS1 and ATCC43504. *Hp*-mediated p53 inactivation and p53 destabilization were verified in our study (Fig. 6A-C and Additional file 1: Fig. S7A). We next examined the effect of *Hp* infection on NAT10 expression. Interestingly, the expression of NAT10 mRNA and protein was significantly induced in the infected cells, and this induction by *Hp* was consistent across several time points (Fig. 6A, C). We then explored the expression of NAT10 in *Hp*-infected mice. When mice were challenged with *Hp* strain SS1, which successfully colonizes the murine stomach [30], they showed a significant induction of NAT10 mRNA and protein expression in the gastric tissues compared with that in control mice (Fig. 6D, E and Additional file 1: Fig. S7B).

In addition, we analyzed the global levels of ac4C modification and MDM2 ac4C levels in *Hp*-infected cells. Consistently, global ac4C modification and MDM2 ac4C levels were increased in GES1 and AGS cells infected with *Hp* (Fig. 6F, G), and MDM2 mRNA stability was enhanced accordingly (Fig. 6H), leading to MDM2 overexpression (Fig. 6A, C). Moreover, the upregulation of MDM2 and the downregulation of p53 and p21 were further confirmed in the *Hp*-infected mice described above (Fig. 6D, E). To verify that *Hp*-mediated p53 downregulation is dependent on NAT10, NAT10-knockout AGS cells and control cells were cocultured with *Hp* and then analyzed for MDM2/p53 expression. As expected, the regulatory effect of *Hp* on MDM2 and p53 expression was reversed by NAT10 knockout (Fig. 6I). Overall, the results demonstrate that *Hp* infection induces the degradation of p53 at least partly via a NAT10-mediated mechanism.

### Targeting NAT10 with Remodelin elicits antitumor activity and improves the sensitivity of GC cells to MDM2 inhibitors

**Fig. 7:**
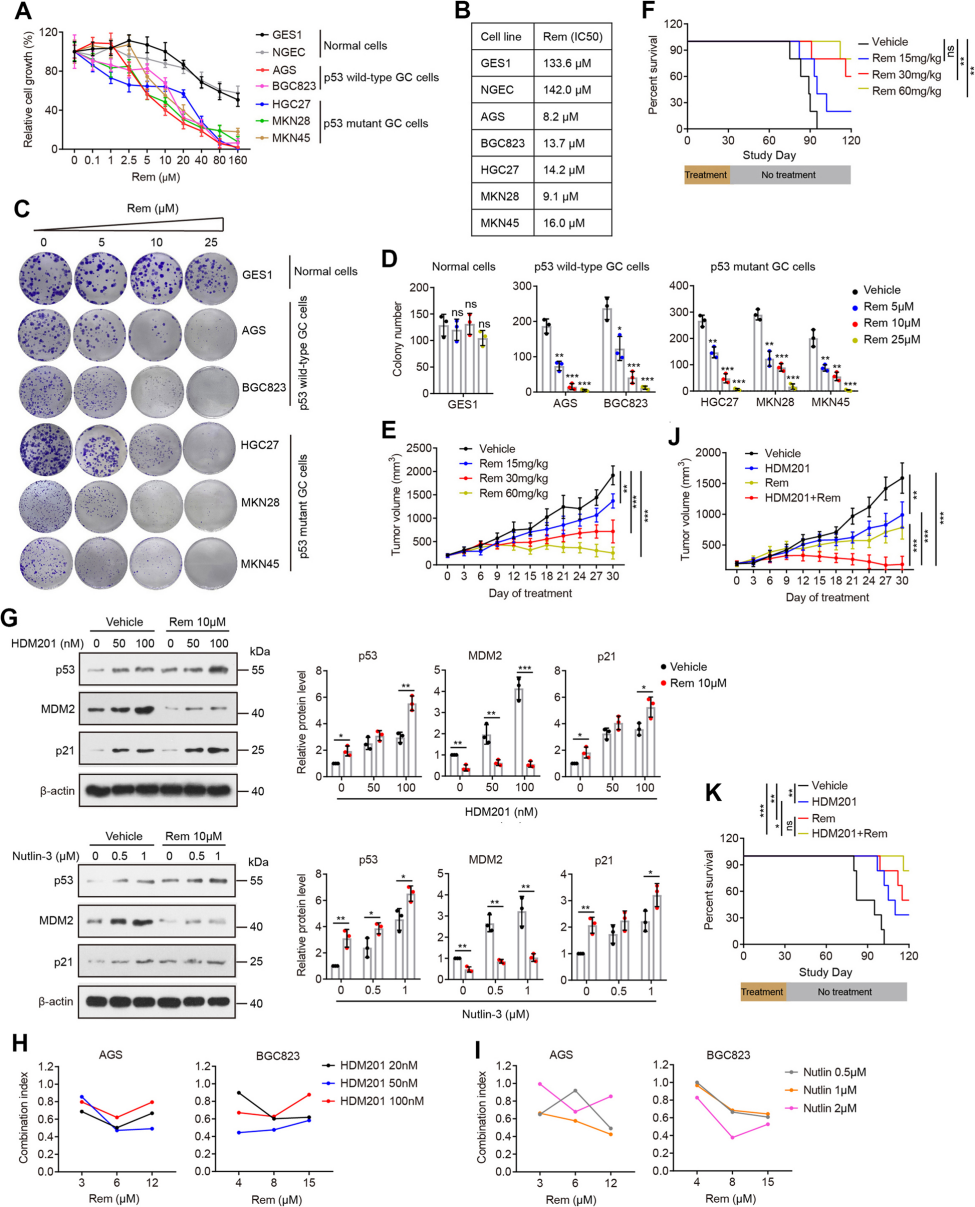
Remodelin suppresses gastric cancer growth and improves the sensitivity of GC cells to MDM2 inhibitors. **A** and **B** Dose–response curves (**A**) and growth IC50 values (**B**) of gastric cancer and normal gastric cell lines following exposure to Remodelin for 72 h in a CCK-8 assay. **C** and **D** Clonogenic assay of gastric cell lines after exposure to increasing concentrations of Remodelin. **E** Nude mice were subcutaneously implanted with BGC823 cells and treated intraperitoneally with different doses of Remodelin daily for 4 weeks (5 mice per group). Tumor volumes were measured as a surrogate for tumor burden. **F** Survival curve of mice treated as described in E. **G** p53, p21 and MDM2 protein levels in AGS cells treated for 24 h with DMSO (vehicle), HDM201 (or Nutlin-3), Remodelin, or a combination of HDM201 (or Nutlin-3) and Remodelin were tested by Western blotting. Densitometric analysis for p53, p21, and MDM2 is shown in the right panel. **H** and **I** Combination study of the inhibitory effects of Remodelin and MDM2 inhibitors on the growth of the p53 wild-type GC cell lines AGS and BGC823 based on a CCK-8 assay. The combination index (CI) values were determined with CompuSyn software. **J** BGC823 cells were subcutaneously implanted into nude mice. Animals were treated daily with vehicle control, HDM201 (10 mg/kg), Remodelin (30 mg/kg), or the combination of both drugs for 4 weeks (6 mice per group). The tumor volume is shown. **K** Survival curves of mice treated in J. Rem, Remodelin; Nutlin, Nutlin-3. Error bars indicate the SD. *P < 0.05, **P < 0.01, ***P < 0.001(two-tailed t-test). ns, not significant

In view of the above results, we subsequently evaluated the anticancer activity of the NAT10 inhibitor Remodelin. We started to evaluate the effect of Remodelin on cell proliferation in a panel of GC cell lines. Remodelin treatment of GC cell lines expressing either wild-type or mutant p53 for 72 h showed a potent inhibitory effect on cell proliferation in a growth IC_50_ range of 8–16 µM (Fig. 7A, B). Remodelin treatment also showed a dose-dependent inhibition of clonogenic growth and induction of apoptosis in both p53 wild-type GC cell lines and p53 mutant GC cell lines (Fig. 7C, D and Additional file 1: Fig. S8). Interestingly, the normal gastric epithelial cell lines GES1 and NGEC were resistant to Remodelin treatment (Fig. 7A-D).

Considering the potent effects of Remodelin in cell culture, we examined the in vivo antitumor properties of Remodelin in mice using subcutaneous xenografts of BGC823 cells. Tumor-bearing mice were treated with vehicle or different doses of Remodelin via intraperitoneal injection. With 60 mg per kg doses (daily), complete tumor growth inhibition was observed in mice (Fig. 7E), and no significant adverse effects, such as weight loss or treatment-related mortality, were observed during Remodelin treatment (Additional file 1: Fig. S9). We also observed that mice treated with Remodelin exhibited increased survival (Fig. 7F).

Given previous reports that treatment with MDM2 inhibitors (inhibitors of the MDM2-p53 interaction) leads to the accumulation of MDM2 as a p53 target following p53 induction (which forms a feedback loop with p53) [31–33], we tested whether Remodelin could potentiate the antitumor effect of MDM2 inhibitors. As expected, treatment with HDM201, a highly selective, orally available active small-molecule MDM2 inhibitor in AGS cells, caused a dose-dependent induction of p53 and p21 expression as well as MDM2 accumulation (Fig. 7G). The combination of HDM201 and Remodelin resulted in higher p53 accumulation and dramatic MDM2 downregulation compared to HDM201 alone, which correlated with stronger activation of the p53 downstream target p21 (Fig. 7G). We subsequently evaluated the antiproliferative effect of combined treatment with HDM201 and Remodelin in GC cell lines and observed remarkable synergistic growth inhibitory activity, i.e., combination index < 1, at most or all concentrations tested in GC cell lines harboring wild-type p53 (Fig. 7H), while p53-mutant cells were resistant to MDM2 inhibitors (Additional file 1: Fig. S10). Similar results were obtained when combining Remodelin and an additional MDM2 inhibitor Nutlin-3 (Fig. 7G, I and Additional file 1: Fig. S10). Using a subcutaneous xenograft model of BGC823 cells, we found that combined administration of HDM201 and Remodelin was significantly more effective in suppressing in vivo tumor growth than either Remodelin or HDM201 alone (Fig. 7J). This correlated with a significant improvement in the survival of mice bearing tumors in the combination treatment group compared with the HDM201-alone treatment group (Fig. 7K). The combination treatment did not induce severe toxicity in mice because there was no significant loss in the total body weight of nude mice bearing tumors (Additional file 1: Fig. S9) or no treatment-related mortality. Together, these data demonstrate that pharmacological inhibition of NAT10 may provide a promising treatment for gastric cancer.

## Discussion

**Fig. 8:**
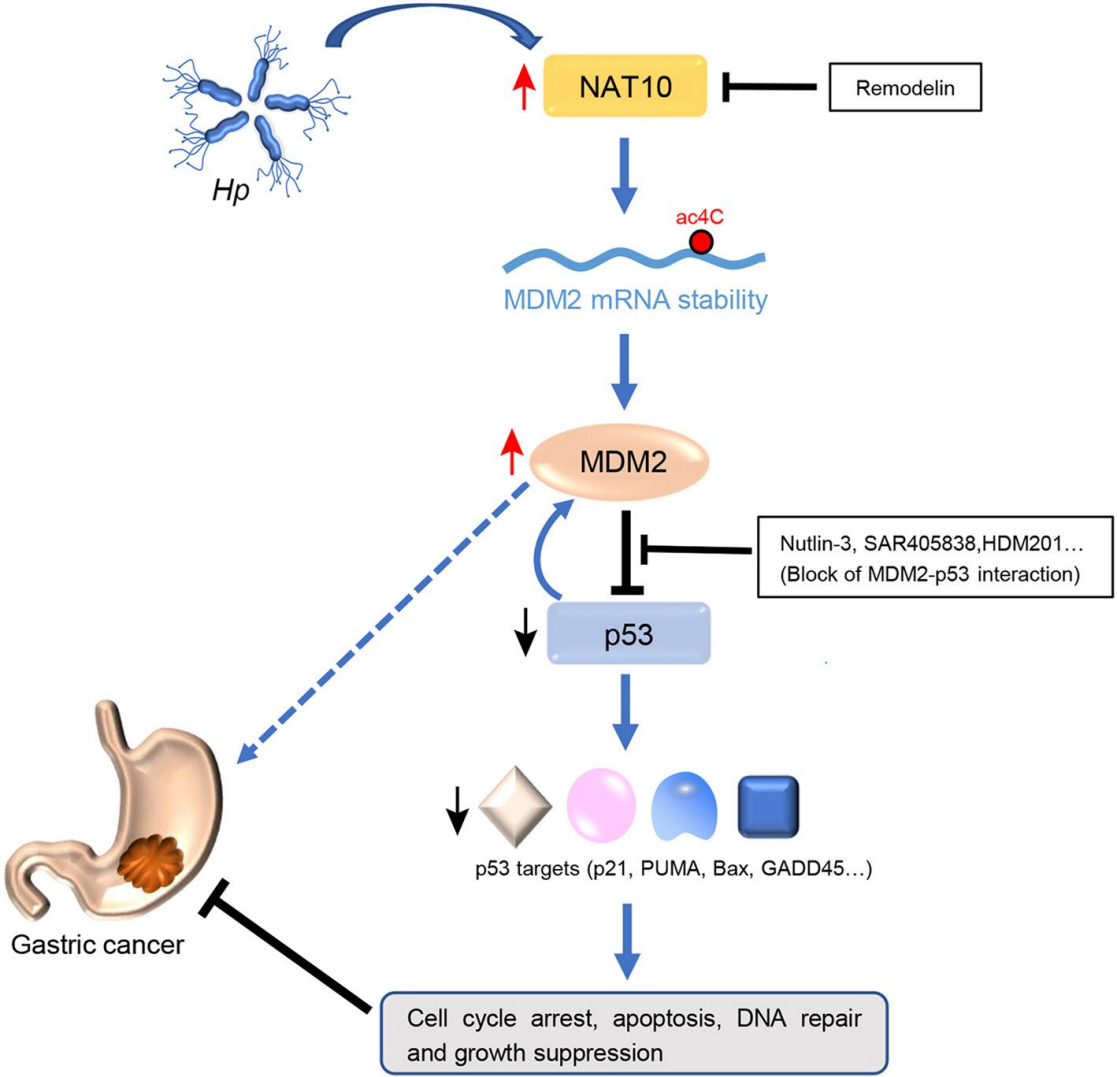
Schematic model for the *Hp*-NAT10-MDM2-p53 axis in promoting the development of GC

ac4C was initially detected on tRNA [18, 34] and rRNA [35], and in recent years, it was also discovered to be widely present in human and yeast mRNAs [8, 9]. However, the biological functions and regulatory impact of ac4C modification remain largely unknown. In the present study, we demonstrated that both the ac4C mRNA modification and its regulator NAT10 are increased in gastric cancer, and increased NAT10 expression correlates with disease progression and poor patient prognosis. We functionally confirmed that NAT10 promotes G2/M transition, proliferation and tumorigenesis in GC cells, and these oncogenic functions rely on its ac4C activity. Mechanistically, NAT10 mediates ac4C modification of MDM2 mRNA to sustain mRNA stability, resulting in MDM2 overexpression, while its own expression is induced by *Hp* infection; MDM2 overexpression contributes to p53 degradation and facilitates the development of GC (Fig. 8).

NAT10 is a member of the GCN5-related N-acetyltransferase (GNAT) family of acetyltransferases. It has been described as a protein acetyltransferase that acetylates target proteins, including histones [36], a-tubulin [19], MORC2 [37], p53[23] and Che-1[38], and as an RNA acetyltransferase that catalyzes the formation of ac4C on rRNA, tRNA and mRNA [39]. Recent studies have shown that deregulation of NAT10 may be involved in the pathogenesis of several types of human cancer. NAT10 was reported to be overexpressed in acute myeloid leukemia and hepatocellular carcinoma and is associated with decreased survival [40, 41]. Moreover, NAT10 could drive liver tumorigenesis in an in vivo steatotic mouse model [41]. NAT10 also confers resistance to DNA-damaging chemotherapy and radiotherapy in breast cancer cells via MORC2 acetylation [37]. However, the exact roles of NAT10, specifically its ac4C-associated biological functions, in human cancers remain undefined. Here, we demonstrated that NAT10 upregulation in GC significantly promotes its growth and dissemination in an ac4C-dependent manner, strongly indicating that NAT10-mediated ac4C modification may be oncogenic.

Our RNA-seq and acRIP-seq high-throughput analyses and subsequent validation and functional studies suggest that MDM2, a well-established oncogene, is the key target of NAT10. NAT10 mediates ac4C modification of MDM2 at its 3′UTR which, in turn, increases its stability, thereby leading to the upregulation of MDM2 and the downregulation of p53. These results are further supported by data showing that *Hp* infection enhances MDM2 ac4C modification and mRNA stability and promotes p53 degradation by inducing NAT10 expression, although further study is required to elucidate the underlying mechanisms by which *Hp* infection impacts NAT10 expression. Notably, a recent report showed that NAT10 mediates ac4C modification on COL5A1 mRNA 3′UTR, which stabilizes the latter in GC cells [42]. However, this is not supported by our acRIP-seq and RNA-seq data in which NAT10 neither regulates ac4C modification on COL5A1 mRNA nor controls its mRNA level.

Amplification and overexpression of the MDM2 gene have been characterized in various cancers [43], and high MDM2 expression is usually linked with higher patient mortality[44]. The oncogenic function of MDM2 is mainly attributed to its ability to target p53 for degradation through binding to p53 [22]. MDM2 overexpression in cancers impairs the functions of wild-type p53, such as DNA repair, cell cycle arrest and apoptosis, and contributes to accelerated tumor growth and progression. Recent studies also indicate that its oncogenic activities extend beyond the regulation of p53 [45]. These characteristics make MDM2 a promising therapeutic target for human cancers. Our study identified MDM2 as an important target of NAT10 and revealed an ac4C modification-dependent mechanism critical to MDM2 activation in GC, creating the possibility to develop therapeutic strategies against GC by targeting ac4C modifications and its regulator, NAT10. Indeed, we observed that the NAT10 inhibitor Remodelin reduces MDM2 ac4C modification and its expression levels and restores wild-type p53 expression. More importantly, it impedes the tumor growth of GC cells harboring either wild-type or mutant p53, which highlights the clinical potential of NAT10 as a drug target in GC. Recently, increasing evidence has suggested that MDM2 has p53-independent activities that can contribute to oncogenesis by interacting with other proteins that can influence cell proliferation, DNA repair and cell fate [46]. This may explain the antiproliferative effect of Remodelin in p53-mutant GC cells.

Here, we further revealed an obvious synergy between Remodelin and MDM2 inhibitors in GC cells with wild-type p53. Disruption of the MDM2-p53 interaction with small-molecule inhibitors of MDM2 for restoring p53 function has been considered as an attractive therapeutic strategy for cancers with wild-type p53 for decades. A number of such small-molecule inhibitors have been developed, and at least seven have recently been advanced into clinical trials [47]. MDM2 inhibitors have demonstrated antitumor activity both in vitro and in vivo in multiple studies. Nevertheless, like other targeted agents, they have limited efficacy in cancer treatment due to primary and acquired resistance [48–50]. In our study, we found that feedback accumulation of MDM2 induced by MDM2 inhibitors is strongly impaired by the combination treatment with Remodelin, causing higher p53 expression in p53 wild-type GC cells, which can enhance the antitumor efficacy of MDM2 inhibitors in the treatment of GC harboring wild-type p53.

## Conclusions

In conclusion, our studies demonstrated the novel ac4C-dependent function of NAT10 in promoting malignant progression of GC through mediating ac4C acetylation of MDM2 mRNA and uncovered a previously unrecognized signaling cascade involving the *Hp*-NAT10-MDM2-p53 axis during GC development. Moreover, our work suggests that targeting NAT10 with Remodelin might be a viable strategy for the treatment of GC with NAT10 overexpression, regardless of p53 status. It is conceivable that the NAT10 inhibitor Remodelin may be a promising drug for GC treatment because it has been characterized [19].

## Supplementary Information

**Additional file 1: Fig. S1.** Levels of multiple mRNA modifications and NAT10 expression in gastric cancer. **Fig. S2.** NAT10 ablation decreases ac4C modification level. **Fig. S3.** Depletion of NAT10 suppresses proliferation, growth and invasion and induces G2/M arrest of gastric cancer cells. **Fig. S4.** Immunohistochemistry analysis of NAT10 and Ki-67 levels in mouse tumors. **Fig. S5.** Characterization of downstream targets of NAT10 via RNA-seq and acRIP-seq assays. **Fig. S6.** Inhibition of NAT10 reduces MDM2 ac4C modification and MDM2 mRNA stability. **Fig. S7.** Verification of *Hp* infection by PCR analysis of *Hp* DNA. **Fig. S8.** Effect of Remodelin on gastric cell apoptosis. **Fig. S9.** Effect of Remodelin and HDM201 on body weight of mice. **Fig. S10.** Combinatorial effects of Remodelin and MDM2 inhibitors on the proliferation of p53-mutant GC cells. **Table S1.** Clinicopathological characteristics of TMA samples. **Table S2.** Correlation between clinicopathological parameters and NAT10 levels in 202 GC tissues (χ^2^-test). **Table S4.** Primers used for qRT-PCR.

**Additional file 2: Table S3.** Differential peaks within mRNA transcipts between NAT10 KO and the control cells.

## Data Availability

RNA-seq and acRIP-seq data have been deposited in the GEO database (GSE180422, https://www.ncbi.nlm.nih.gov/geo/query/acc.cgi?acc=GSE180422, and GSE180494, https://www.ncbi.nlm.nih.gov/geo/query/acc.cgi?acc=GSE180494). The other data from the authors are available upon reasonable request. RNA-seq data from the TCGA stomach adenocarcinoma (STAD) dataset were downloaded from the Genomic Data Commons (GDC) data portal (https://gdc.cancer.gov/developers/gdc-application-programming-interface-api). Gene expression array data were downloaded from the GEO database (GSE29272, https://www.ncbi.nlm.nih.gov/geo/query/acc.cgi?acc=GSE29272, and GSE66229, https://www.ncbi.nlm.nih.gov/geo/query/acc.cgi?acc=GSE66229).

## Abbreviations

GC: Gastric cancer
ac4C: N4-acetylcytidine
m5C: 5-Methylcytidine
m1A: N1-methyladenosine
Am: 2’-O-methyladenosine
m6A: N6-methyladenosine
NAT10: N-acetyltransferase 10
*Hp*: *Helicobacter pylori*
mRNA: Messenger RNA
HPLC–MS/MS: High-performance liquid chromatography-mass spectrometry/mass spectrometry
TCGA: The Cancer Genome Atlas
GEO: Gene expression omnibus
qRT-PCR: Quantitative reverse transcription polymerase chain reaction
IHC: Immunohistochemistry
shRNA: Short hairpin RNA
RIP: RNA immunoprecipitation
acRIP-seq: Ac4C RNA immunoprecipitation-sequencing
RNA-seq: RNA sequencing
TSS: Transcription start site
UTR: Untranslated region
CDS: Coding sequence
KO: Knockout
GO: Gene Ontology
KEGG: Kyoto Encyclopedia of Genes and Genomes
OS: Overall survival
FP: First progression
PPS: Post progression survival
