# Correction: *Helicobacter pylori*-induced NAT10 stabilizes MDM2 mRNA via RNA acetylation to facilitate gastric cancer progression

**DOI:** 10.1186/s13046-023-02676-3

**Published:** 2023-04-24

**Authors:** Min Deng, Long Zhang, Wenying Zheng, Jiale Chen, Nan Du, Meiqi Li, Weiqing Chen, Yonghong Huang, Ning Zeng, Yuanbin Song, Yongming Chen

**Affiliations:** 1 Afliated Cancer Hospital & Institute of Guangzhou Medical University, Guangzhou Key Laboratory of “Translational Medicine On Malignant Tumor Treatment”, Guangzhou, 510095 China; 2 Sun Yat-Sen University Cancer Center, State Key Laboratory of Oncology in South China, Collaborative Innovation Center for Cancer Medicine, Guangzhou, 510060 China; 3 First Department of Hepatobiliary Surgery, Zhujiang Hospital, Southern Medical University, Guangdong Provincial Clinical and Engineering Technology Center of Digital Medicine, Guangzhou, 510280 China

**Correction: J Exp Clin Cancer Res 42, 9 (2023)**

**https://doi.org/10.1186/s13046-022-02586-w**

Following publication of the original article [1], an error was identified in Fig. 4, specifically:

- Figure 4D - AGS/CTR and AGS/KO+MDM2 have been uploaded repeatedly

Correct figure is presented below:

**Fig. 4:**
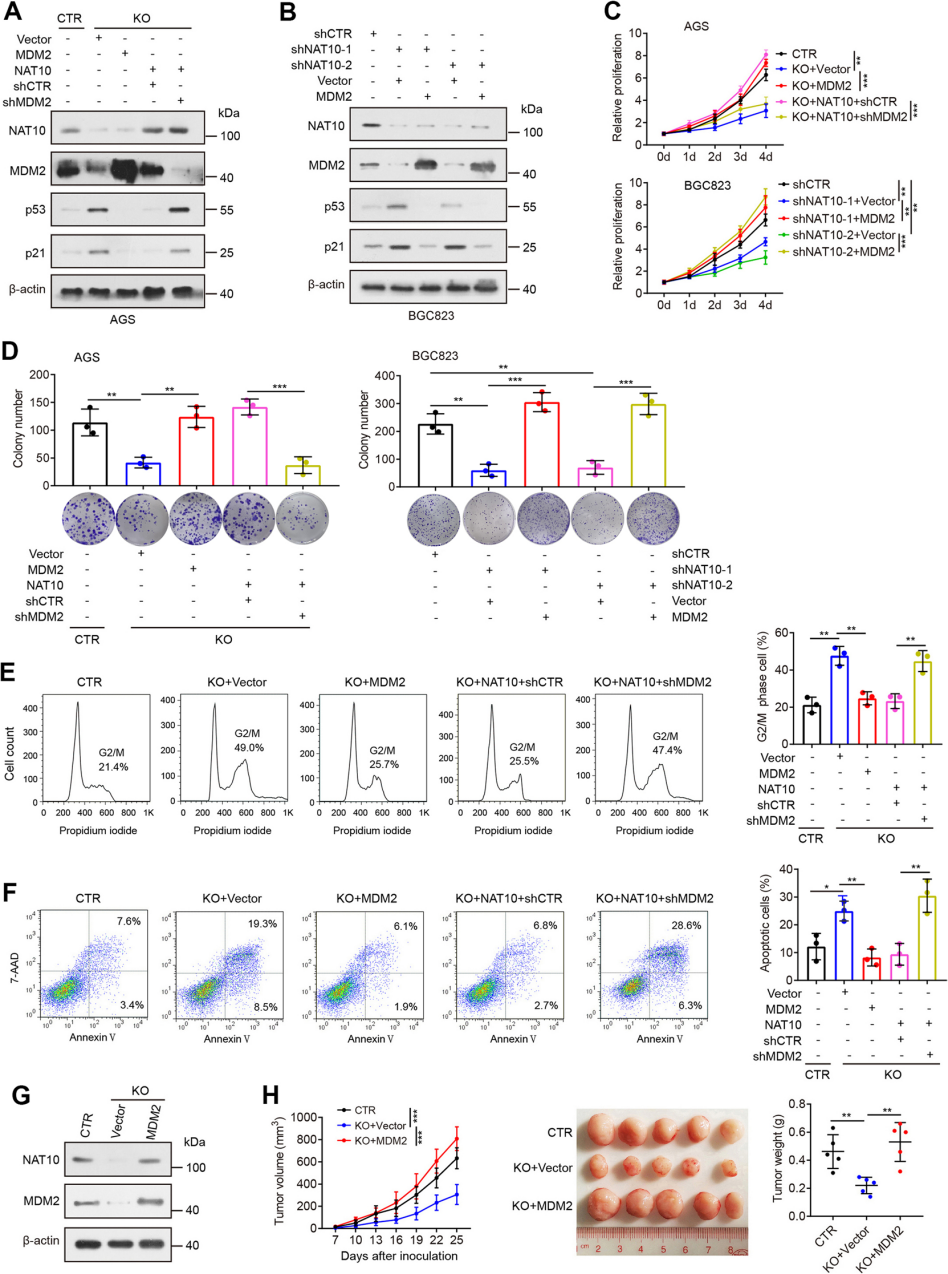
MDM2 is a major contributor to the function of NAT10 in gastric carcinogenesis. **A** Overexpression of MDM2 inhibited the upregulation of p53 and p21 proteins in NAT10-knockout AGS cells, while knockdown of MDM2 efectively reversed the inhibitory efect of NAT10 overexpression on p53 and p21. **B** MDM2 overexpression reversed the upregulation of p53 and p21 proteins by NAT10 knockdown in BGC823 cells. **C** and **D** The efects of NAT10 depletion on cell proliferation (**C**) and colonic growth (**D**) were rescued by transfection with MDM2, whereas cell proliferation and colonic growth of NAT10-overexpressing cells were prevented by knockdown of MDM2. **E** and **F** Cell cycle (**E**) and apoptosis (**F**) were measured in the indicated cells by fow cytometry. **G** MDM2 and NAT10 proteins were evaluated in NAT10-knockout AGS cells stably expressing MDM2 or vector control. **H** MDM2 overexpression rescued the impaired capacity of tumor growth triggered by NAT10 knockout (*n*=5 mice/group). Error bars, SD. *P<0.05, **P<0.01, ***P<0.001 using a two-tailed t-test
