# Supplementary material for: Helicobacter pylori-induced NAT10 stabilizes MDM2 mRNA via RNA acetylation to facilitate gastric cancer progression
Source: J Exp Clin Cancer Res. 2023 Jan 6;42:9. doi: 10.1186/s13046-022-02586-w (PMC9817303; doi:10.1186/s13046-022-02586-w)
Supplement: Supplementary file 1 — Additional file 1: Fig. S1. Levels of multiple mRNA modifications and NAT10 expression in gastric cancer. Fig. S2. NAT10 ablation decreases ac4C modification level. Fig. S3. Depletion of NAT10 suppresses proliferation, growth and invasion and induces G2/M arrest of gastric cancer cells. Fig. S4. Immunohistochemistry analysis of NAT10 and Ki-67 levels in mouse tumors. Fig. S5. Characterization of downstream targets of NAT10 via RNA-seq and acRIP-seq assays. Fig. S6. Inhibition of NAT10 reduces MDM2 ac4C modification and MDM2 mRNA stability. Fig. S7. Verification of Hp infection by PCR analysis of Hp DNA. Fig. S8. Effect of Remodelin on gastric cell apoptosis. Fig. S9. Effect of Remodelin and HDM201 on body weight of mice. Fig. S10. Combinatorial effects of Remodelin and MDM2 inhibitors on the proliferation of p53-mutant GC cells. Table S1. Clinicopathological characteristics of TMA samples. Table S2. Correlation between clinicopathological parameters and NAT10 levels in 202 GC tissues (χ2-test). Table S4. Primers used for qRT-PCR. [file 13046_2022_2586_MOESM1_ESM.docx]

**Supplementary Materials and Methods**

**Antibodies**

The following antibodies were used in this study: anti-N4-acetylcytidine (ac4C), rabbit monoclonal, Abcam (ab252215); anti-NAT10, rabbit monoclonal, Abcam (ab194297); anti-MDM2, mouse monoclonal, Abnova (H00004193-M01); anti-p53, mouse monoclonal, BD Biosciences (610183); anti-p53 (acetyl K120), mouse monoclonal, Abcam (ab78316); anti-p21, rabbit monoclonal, Cell Signaling Technology (2947); anti-PUMA, rabbit monoclonal, Cell Signaling Technology (98672); anti-β-actin, mouse monoclonal, Proteintech Group (66009-1-Ig); anti-Ki-67, mouse monoclonal, Cell Signaling Technology (9449); anti-GAPDH, rabbit monoclonal, Cell Signaling Technology (5174); anti-rabbit IgG (HRP), Invitrogen (65-6120); anti-mouse IgG (HRP), Invitrogen (62-6520); anti-rabbit IgG, Sigma-Aldrich (NI01).

**HPLC–MS/MS analysis of RNA modifications**

HPLC–MS/MS analysis of RNA modifications was adapted from a previously reported protocol [1]. Briefly, total RNA was eluted using TRIzol reagent (Invitrogen), and mRNA was then purified from total RNA using biotin-oligo (dT) streptavidin beads from Promega following the corresponding manufacturer’s recommendations. One microgram of total RNA or mRNA was digested by nuclease S1, phosphodiesterase and alkaline phosphatase and then analyzed using a UPLC-ESI-MS/MS system. The nucleosides were separated by reversed-phase ultra-performance liquid chromatography (ExionLC™ AD) on a C18 column (1.8 μm, 100 mm×2.1 mm i.d.) and detected in positive ion multiple reaction-monitoring (MRM) mode using ESI-triple quadrupole-linear ion trap (QTRAP)-MS (Applied Biosystems 6500 Triple Quadrupole). RNA modifications were qualified with the standard curves calculated from pure nucleoside standards run concurrently with the samples.

**Cell culture**

The human GC cell lines BGC823, SGC7901, HGC27, MKN28, MKN45 and MGC803 were obtained from the Chinese Academy of Medical Science (Beijing, China), and AGS, SNU1 and NCI-N87 cells were obtained from ATCC. The murine GC cell line MFC was obtained from Procell Life Science & Technology (Wuhan, China). The human gastric epithelial cell lines GES1 and NGEC were obtained from the Beijing Institute for Cancer Research (China) and Honsun Biological Technology (Shanghai, China), respectively. The HEK293T cell line was purchased from ATCC. Cell lines involved in our experiments were reauthenticated via STR genotyping every 6 months after resuscitated. Cells were maintained in RPMI 1640 medium (Gibco) supplemented with 10% FBS (Gibco).

**ac4C dot blot assay**

The ac4C dot blot assay was performed as previously described [2]. Briefly, the mRNA samples were loaded onto nitrocellulose membranes (Millipore-Merck) with a Bio-Dot Apparatus (Bio–Rad) and crosslinked with ultraviolet (UV) radiation. The membranes were blocked with 5% nonfat milk and probed with anti-ac4C followed by HRP-linked anti-rabbit IgG antibodies. Immunoreactive dots were visualized by developing the membranes by chemiluminescence (Pierce).

**qRT–PCR**

Total RNA was reverse-transcribed with the QuantiTect Reverse Transcription kit (Qiagen), and subsequent qPCR was performed using SYBR-green reagents (Applied Biosystems). Gene expression was normalized to GAPDH or ACTB. The primers used are listed in Additional file 1: Table S4.

**Western blot**

Total protein was isolated with RIPA buffer (Beyotime Biotechnology) containing phosphatase inhibitor (Thermo Scientific). Proteins were separated by 10% SDS-PAGE, and transferred to PVDF membranes (Millipore-Merck). The membranes then probed with primary and secondary antibodies after blocked in 5% nonfat milk in TBST. They were then washed with TBST, and the blots were visualized with a commercial ECL kit (Pierce).

**Immunohistochemistry**

Immunohistochemistry analysis was conducted as previously described [3]. The NAT10 and MDM2 expression levels in human tissues were determined based on the staining intensity and percentage of cells with positive NAT10 or MDM2 staining. The intensity of staining was scored from 0 to 3 (0, no staining; 1, weak; 2, moderate; 3, strong), and the percentage of positive staining was scored from 0 to 4 (0, <5% positive staining; 1, 5–24% positive staining; 2, 25–50% positive staining; 3, 51–75% positive staining; 4, >75% positive staining). The final score was obtained by multiplying the two scores. The cutoff value for low or high expression of the two proteins was a final score of <6.

**Plasmid and RNAi**

The full-length NAT10 CDS and NAT10 Δhelicase (lacking amino acids 281–488) were chemically synthesized and then cloned into the pEZ-M56 mammalian expression vector (GeneCopoeia, Guangzhou, China) with a Flag tag, hereafter named pNAT10 and pΔhelicase, respectively. The constructs containing the K290A and G641E mutations of NAT10 were generated by introducing point mutations with a QuikChange Site-Directed Mutagenesis kit (200518, Stratagene) using the pNAT10 plasmid as a template, termed pK290A and pG641E, respectively. The wild-type MDM2 3′UTR fragment or mutant counterpart (in which the region covered by the ac4C peak was deleted) was inserted into the dual-luciferase reporter vector pmirGLO (Promega), which were named Wt-3′UTR and Mut-3′UTR, respectively. The MDM2 promoter sequence (2 kb of sequence upstream of the TSS) was amplified by PCR and cloned into the pGL3-Basic vector. The recombinant MDM2 expression vector for MDM2 overexpression and recombinant lentiviruses containing shRNAs targeting NAT10 or MDM2 were obtained from GeneCopoeia (Guangzhou, China). The target sequences for NAT10 and MDM2 were as follows: shNAT10-1, 5′-CGGCCATCTCTCGCATCTATT-3′; shNAT10-2, 5′- GCAATTGTACACAGTGACTAT-3′; and shMDM2, 5′-AATACCAACATGTCTGTACCT-3′.

**RNA-seq analysis**

Total RNA from AGS cells with or without NAT10 knockout and BGC823 cells expressing shNAT10-2 or control shRNA was isolated using TRIzol reagent. Poly(A)+ mRNA was subsequently purified from total RNA via a Dynabeads mRNA Purification Kit (61006, Invitrogen) and then sequenced on an Illumina NovaSeq 6000 platform by Epibiotek (Guangzhou, China). The reads were mapped to human genome version 38 (GRCh38) with HISAT2 (v2.1.0) and then calculated using HT-seq (v0.7.2). Genes with a *P* value < 0.05 and fold change > 1.2 were considered to be differentially expressed. The online tool DAVID (www.david.niaid.nih.gov) was used to perform GO enrichment and KEGG pathway analyses.

**acRIP-seq analysis**

acRIP-seq and subsequent data analyses were mainly supported by Epibiotek (Guangzhou, China). Briefly, total RNA from NAT10-knockout or control AGS cells was extracted using TRIzol reagent, digested with DNase I and fragmented into 100~200 nt oligonucleotides by RNA fragmentation reagents (ab252215, Ambion). After saving 50 ng of fragmented RNA as the input, the remainder (150 μg) was used for RNA immunoprecipitation with ac4C antibody (Abcam). ac4C RNAs were immunoprecipitated with Dynabeads Protein G (10004D, Invitrogen) and recovered with HiPure cell miRNA (R4311-03, Magen, Guangzhou, China). Ribosomal RNA was removed from input RNA and ac4C-enriched RNA samples. RNA sequencing libraries for input RNA (RNA-seq) and ac4C-enriched RNA (acRIP-seq) were simultaneously constructed with the EpiTM mini longRNA-seq kit (E1802, Epibiotek) and then deep sequenced on the Illumina NovaSeq 6000 using PE150 strategy with two independent biological replicates. Reads were aligned to the human genome GRCh38/hg38 with HISAT2 software (v2.1.0). Then, ac4C peaks were identified using the ExomePeak R package (v2.13.2). The differential ac4C peaks with fold change > 2.0 and *P* value < 0.05 were selected.

**acRIP-qPCR**

The acRIP procedure was performed according to a previously described protocol with slight modifications.[4] Total RNA was isolated from the indicated cells with additional DNase I on-column digestion. Five micrograms of RNA were incubated with 10 μg of anti-ac4C antibody or anti-rabbit IgG for 2 h at 4 °C. The immune complexes were captured by Protein G magnetic beads (Invitrogen) for 2 h at 4 °C. The RNA of interest was extracted from the immunoprecipitated complex RNA with TRIzol, and ac4C enrichment was determined for qRT–PCR analysis as described above. Relative enrichment of ac4C was normalized to anti-rabbit IgG.

**RIP assay**

RIP was performed by using an Imprint® RNA Immunoprecipitation (RIP) Kit (RIP-12RXN, Sigma-Aldrich). Briefly, NAT10-knockout AGS, NAT10-knockdown BGC823 and their corresponding control cells were lysed in RIP lysis buffer. 20 µl of Protein-A magnetic beads were mixed with 5 μg of anti-NAT10 or anti-rabbit IgG and then the antibody prebound magnetic beads were incubated with the cell lysates at 4 °C for 3 h. Following treatment with proteinase K, RNA was isolated from the immunoprecipitated complex for qRT–PCR analysis. The relative enrichment of NAT10 was normalized to that of anti-rabbit IgG.

**Colony formation and cell proliferation/growth assays**

To test the capacities of cell colony formation, cells were (800–1000 cells per well) seeded in six-well plates and cultured for 9 to 14 days. The colonies were stained with crystal violet and counted manually. For cell growth/proliferation assays, cells (1000 cells/well) were seeded into 96-well plates in triplicate. Cell growth/proliferation was tested using CCK-8 (Promega) at various time points following the manufacturer’s recommendations. Absorbance was assessed at 490 nm (BioTek Synergy2).

**Soft agar colony formation assay**

Six-well plates were coated with a layer of 0.6% agar in medium supplemented with 20% FBS. A total of 3000–5000 cells in 0.3% agar were seeded per well and cultured at 37 °C for 10–14 days.

**Cell invasion assay**

The cells were starved overnight, collected in serum-free medium and then seeded into the upper chamber precoated with Matrigel on a polycarbonate membrane (8.0 µM permeable) in the insert of a 24-well plate (Corning Costar). Then, 700 μl of medium containing 15% FBS was added to the bottom chamber. After culture for 48 to 72 hours at 37 °C, the invading cells on the lower surface of the membrane were stained and photographed.

**Cell apoptosis and cycle assays**

For analysis of apoptosis after specific conditions or treatments, cells were fixed and stained with Annexin V-APC/7-AAD (Multi Science Biotech, Hangzhou, China), and apoptosis was measured using ﬂow cytometry (FACSCalibur instrument, BD Biosciences). To assess the cell cycle, cells were harvested and fixed in 70% ethanol. Then, the cells were stained with propidium iodide and analyzed by ﬂow cytometry.

**Luciferase reporter assay**

The luciferase reporter vector fused with the wild-type or mutant MDM2 3′UTR was transfected into the indicated cells, and the firefly and Renilla luciferase activities were measured with the Dual-Glo Luciferase Assay system (Promega). In addition, NAT10-knockout AGS cells, BGC823 cells expressing NAT10 shRNAs and their corresponding control cells were cotransfected with the MDM2 promoter reporter construct and the pTK-Cluc vector (as an internal control), and the luciferase activities were measured.

**Subcellular fractionation assay**

Subcellular fractionation was carried out with a PARIS Kit (AM1921, Invitrogen). Briefly, cells were collected and incubated with cold Cell Fractionation Buffer for 10 min on ice. The cytoplasmic fraction (the supernatant) and nuclear fraction (the pellet) were separated by centrifuge at 500 g for 2min at 4°C. Then, total RNA was isolated and qRT-PCR analysis was performed.

**Infection of cell lines or mice with *Hp***

*Hp* infection experiment was carried out in biosafety shelter of microbiological laboratory. The *Hp* strains SS1 (Sydney strain 1) and ATCC43504 were obtained from the Institute of Microbiology, Guangdong Academy of Sciences (China). *Hp* bacteria were initially grown on Columbia blood agar plates (Detgerm, Guangzhou, China) and then cultured in Brucella broth (Invitrogen) with 10% FBS for 18 h at 37 °C under microaerobic conditions. For *in vitro* studies, GES1 and AGS cells were infected with *Hp* strains at a bacteria-to-cell ratio of 50:1. To establish a murine *Hp* infection model, *Hp* SS1 (10^9^ CFU per mouse) or Brucella broth were orally gavaged into C57BL/6 mice every other day for a total of 5 doses. At 3 weeks after infection, mice were sacrificed, and gastric tissues were collected for further study.

**PCR quantitation of *Hp* DNA**

DNA was extracted from human cell lines and mouse gastric tissues with a HiPure Tissue DNA Mini kit (Magen, Guangzhou, China). *Hp*-specific 16S ribosomal DNA (rDNA) was detected by semiquantitative PCR or real-time PCR as previously described using specific primers HP5 (5′-TTTGTTAGAGAAGATAATGACGGTATCTAAC-3′) and HP6 (5′-CATAGGATTTCACACCTGACTGACTATC-3′) [5, 6]. Human/mouse GAPDH was measured for normalization. The GAPDH primers were as follows: GAPDH (human), forward, 5ʹ- CCGTTGCACAATACCTTCGG-3ʹ, reverse: 5ʹ-CATTACGCGTAGGGGTTTGA -3ʹ; and GAPDH (mouse), forward: 5ʹ- TAATCACGCGTGGACTGACC-3ʹ, reverse: 5ʹ- GGCCAAGCATCATGCCAAAA -3ʹ.

**References**

1. Basanta-Sanchez M, Temple S, Ansari SA, D'Amico A, Agris PF. Attomole quantification and global profile of RNA modifications: Epitranscriptome of human neural stem cells. Nucleic Acids Res. 2016;44:e26.

2. Li Z, Weng H, Su R, Weng X, Zuo Z, Li C, et al. FTO Plays an Oncogenic Role in Acute Myeloid Leukemia as a N(6)-Methyladenosine RNA Demethylase. Cancer Cell. 2017;31:127-141.

3. Deng M, Zhang R, He Z, Qiu Q, Lu X, Yin J, et al. TET-Mediated Sequestration of miR-26 Drives EZH2 Expression and Gastric Carcinogenesis. Cancer Res. 2017;77:6069-6082.

4. Arango D, Sturgill D, Alhusaini N, Dillman AA, Sweet TJ, Hanson G, et al. Acetylation of Cytidine in mRNA Promotes Translation Efficiency. Cell. 2018;175:1872-1886 e1824.

5. Hu W, Zhang L, Li MX, Shen J, Liu XD, Xiao ZG, et al. Vitamin D3 activates the autolysosomal degradation function against Helicobacter pylori through the PDIA3 receptor in gastric epithelial cells. Autophagy. 2019;15:707-725.

6. Zhang L, Hu W, Cho CH, Chan FK, Yu J, Fitzgerald JR, et al. Reduced lysosomal clearance of autophagosomes promotes survival and colonization of Helicobacter pylori. J Pathol. 2018;244:432-444.

**Supplementary Fig. S1-S10**


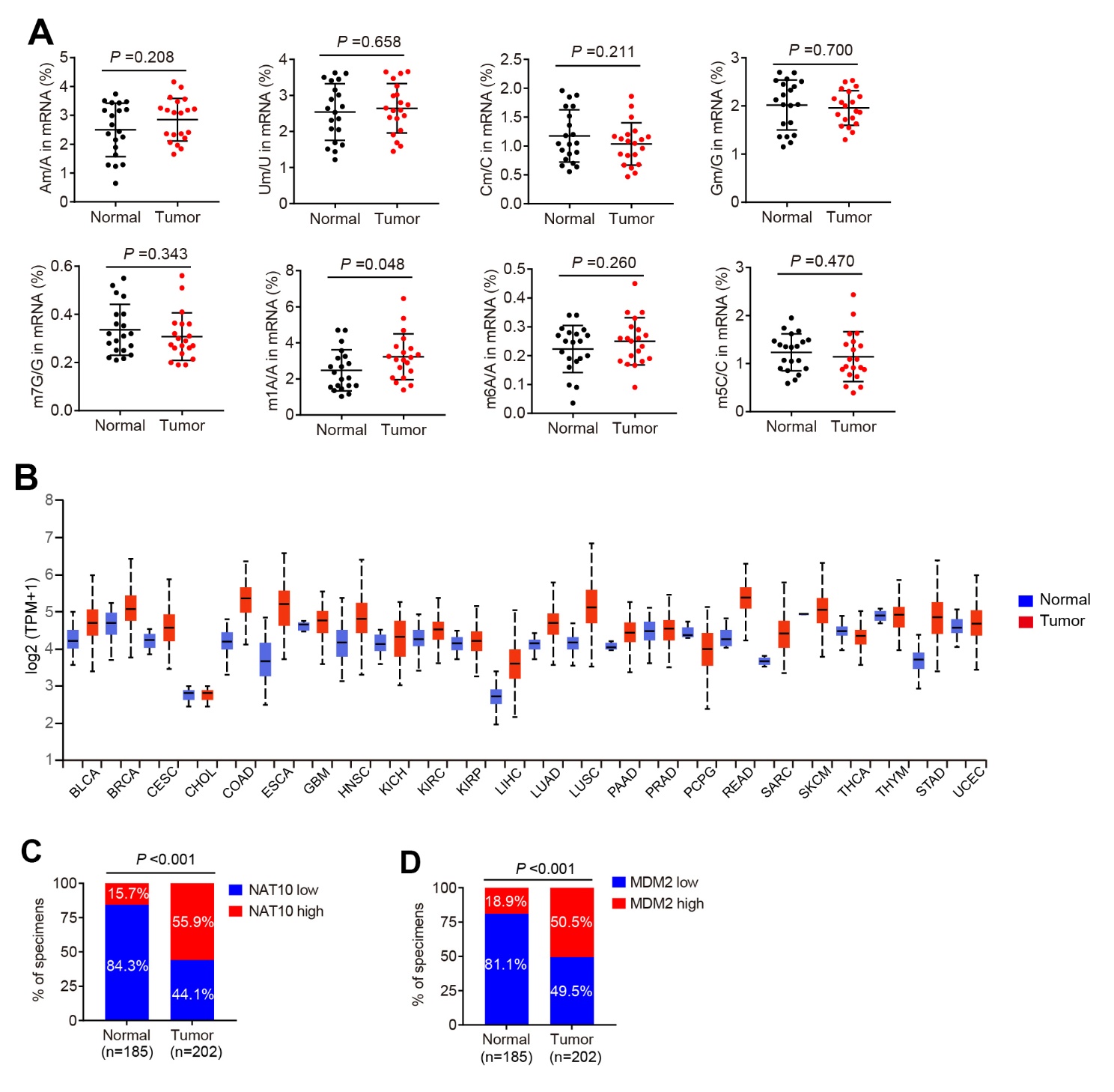


**Fig. S1. Levels of multiple mRNA modifications and NAT10 expression in gastric cancer.** (A) The levels of distinct chemical modifications in polyA (+) mRNA isolated from 20 GCs (Tumor) and paired normal gastric mucosal tissues (Normal) were quantified by HPLC–MS/MS. Error bars, SD. *P* values were calculated with a two-tailed t-test. (B) The expression of NAT10 in various cancers (Tumor) and their corresponding normal tissues (Normal) was analyzed with UALACN (data from TCGA). (C) Quantification of NAT10 IHC staining in normal gastric epithelium and gastric tumors. P value was determined by the χ2-test. (D) Quantification of MDM2 staining in normal gastric epithelium and gastric tumors is plotted in G (χ2-test).


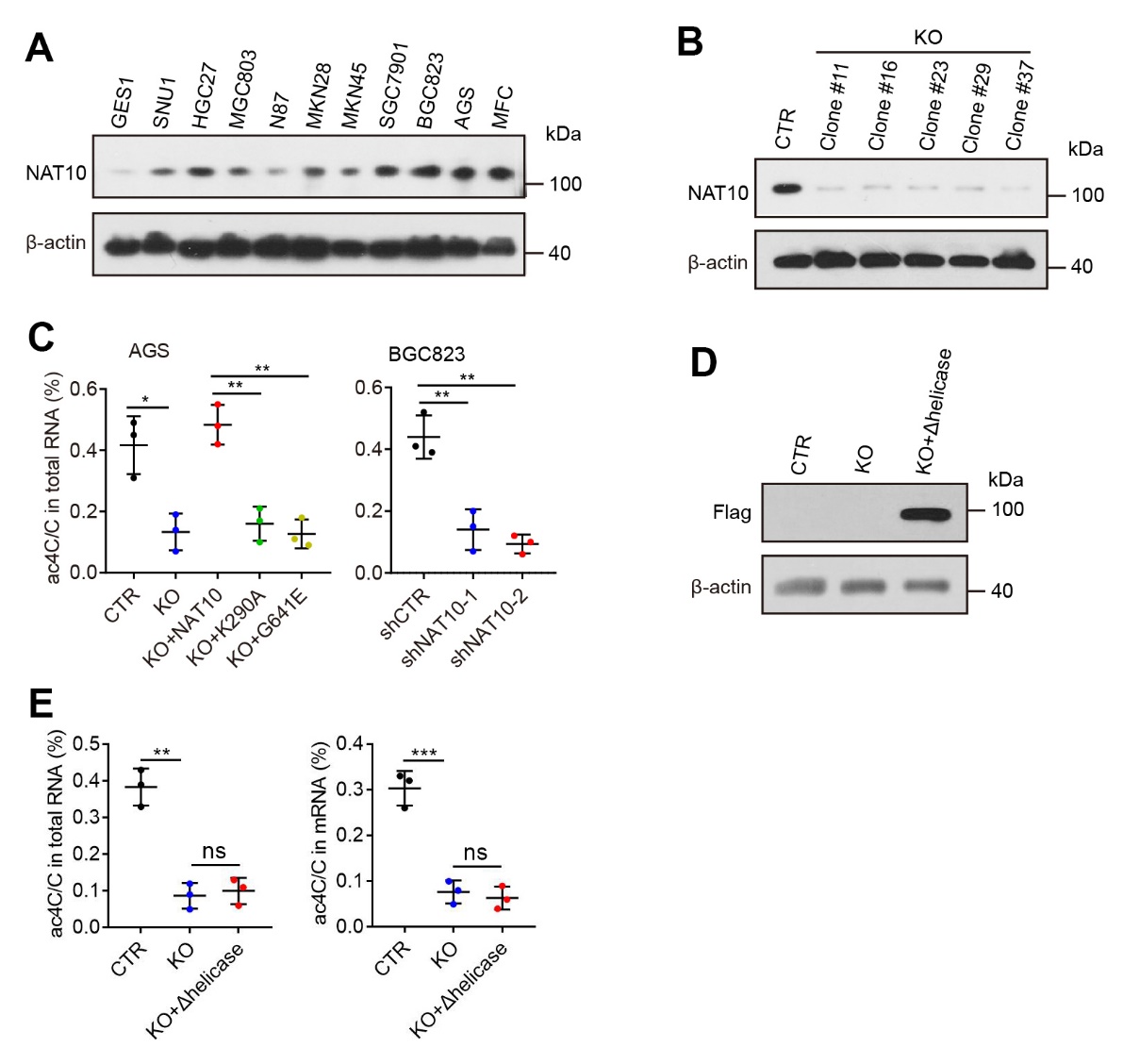


**Fig. S2. NAT10 ablation decreases ac4C modification level.** (A) Western blots showing NAT10 protein levels in GC cell lines and the normal gastric epithelial cell line (GES1). (B) Western blotting was conducted to validate the knockout efficiency in NAT10-knockout cells derived from different clones. (C) HPLC–MS/MS quantification of ac4C levels in total RNA from parental AGS cells, NAT10-knockout cells, knockout cells re-expressing wild-type or mutant NAT10, and BGC823 cells with or without NAT10 shRNAs. (D, E) Western blot analysis (D) and HPLC–MS/MS quantification of ac4C levels in total RNA and mRNA (E) were performed with control AGS cells, NAT10-knockout cells, and knockout cells transfected with Flag-tagged NAT10 Δhelicase. Error bars represent SDs from three independent experiments. **P* < 0.05, ***P* < 0.01, ****P* < 0.01. ns, not significant.


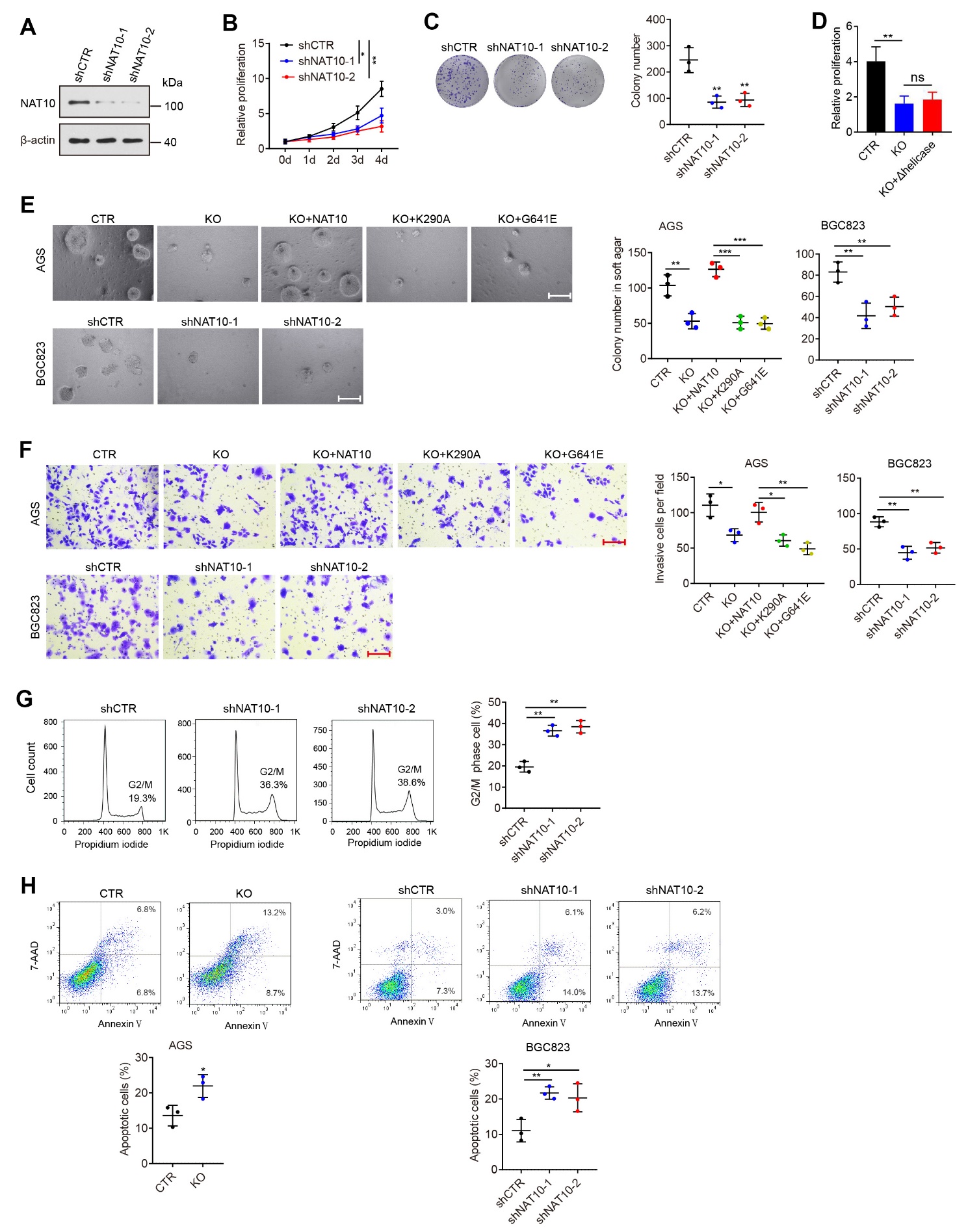


**Fig. S3. Depletion of NAT10 suppresses proliferation, growth and invasion and induces G2/M arrest of gastric cancer cells.** (A-C) Western blot, CCK-8 proliferation and colony formation assays in MKN45 cells transfected with control shRNA or NAT10 shRNAs. (D) The control AGS cells, NAT10-knockout cells, and knockout cells transfected with NAT10 Δhelicase were seeded into 96-well plates, and cell proliferation was tested with CCK-8 after 3 days of incubation. (E, F) Soft agar colony formation (E) and cell invasion (F) assays were carried out in control AGC cells, NAT10-knockout cells, knockout cells re-expressing wild-type NAT10 or mutant NAT10, and BGC823 cells with or without NAT10 shRNAs. (G) Cell cycle analysis in BGC823 cells with or without NAT10 knockdown. (H) Apoptosis was examined in NAT10-knockout AGS cells, BGC823 cells with NAT10 knockdown and their corresponding controls. Error bars indicate SDs from three independent experiments. **P* < 0.05, ***P* < 0.01, ****P* < 0.001 (two-tailed t-test).


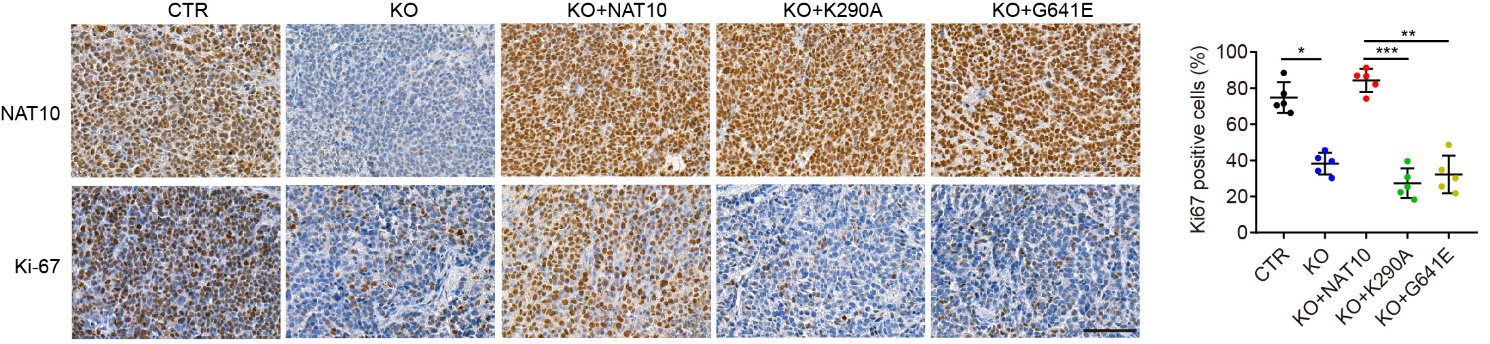


**Fig. S4. Immunohistochemistry analysis of NAT10 and Ki-67 levels in mouse tumors.** Scale bar, 100 μm. Error bars, SD. **P* < 0.05. ***P* < 0.01, ****P* < 0.001 (two-tailed t-test).


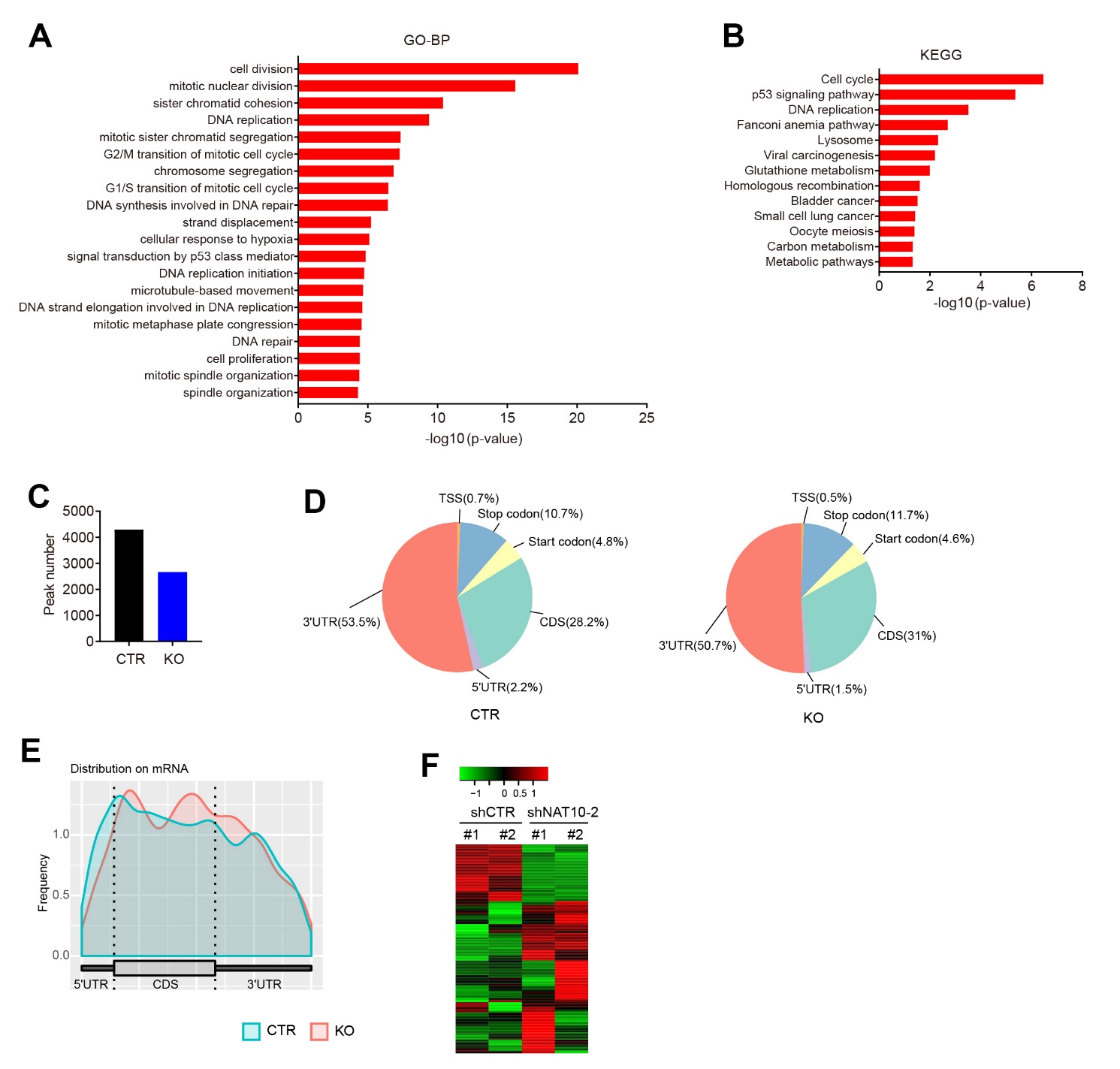


**Fig. S5.** **Characterization of downstream targets of NAT10 via RNA-seq and acRIP-seq assays.** (A) Top 20 significantly affected biological processes (BPs) by GO enrichment analysis of the differentially expressed genes in NAT10-knockout vs. control cells. (B) KEGG pathway analysis was conducted with the differentially expressed genes identified between NAT10-knockout and control cells. (C) Number of ac4C peaks identified in acRIP-seq in NAT10-knockout and control cells. (D) Pie chart showing the proportion of ac4C peak distribution in the different regions within mRNA transcripts. (E) Metagene plots showing the distribution of ac4C peaks identified across mRNA transcripts in NAT10-knockout and control cells. (F) Heat map showing the differentially expressed genes in NAT10-knockdown vs. control BGC823 cells.


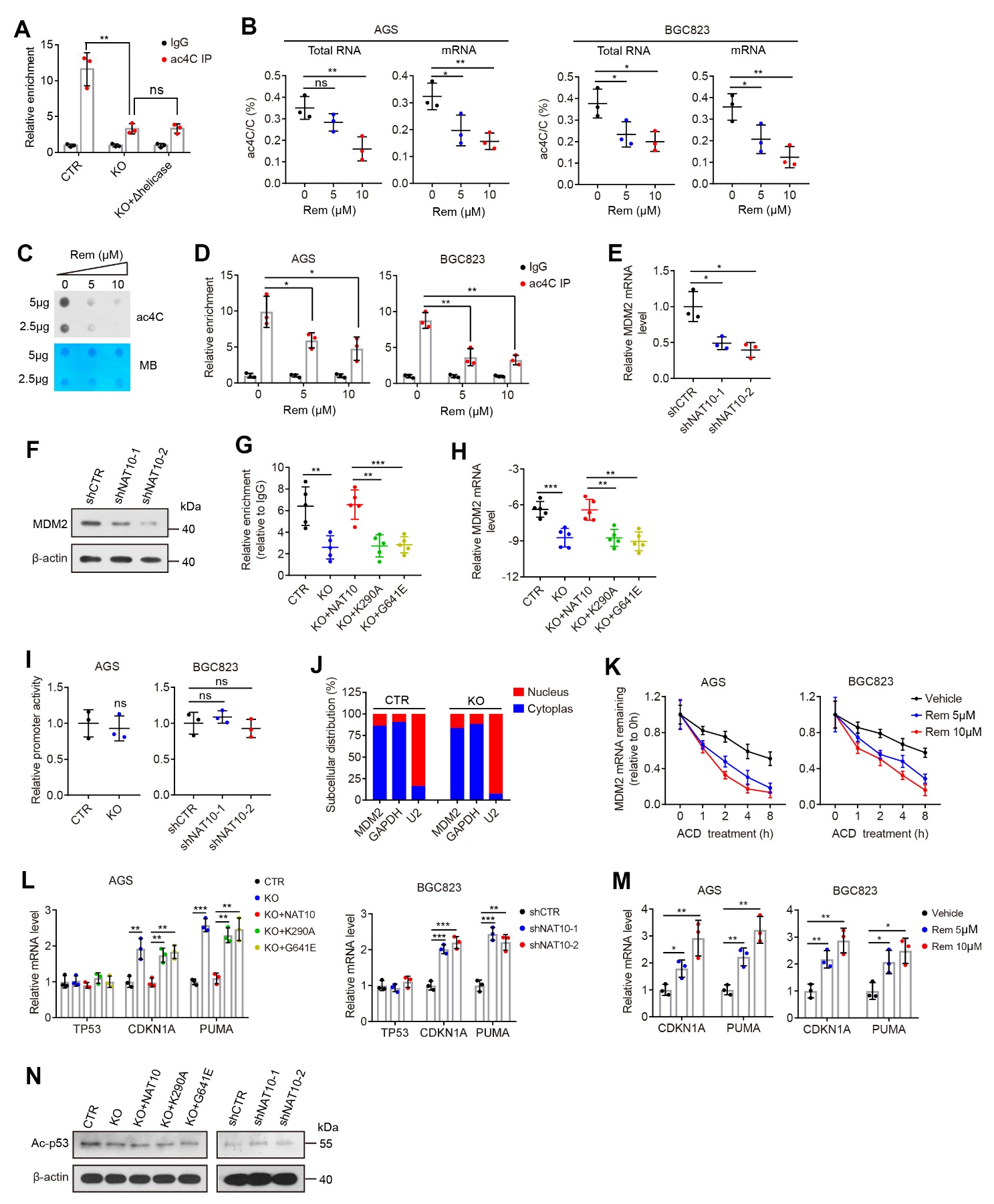


**Fig. S6. Inhibition of NAT10 reduces MDM2 ac4C modification and MDM2 mRNA stability.** (A) The relative ac4C levels of MDM2 mRNA in control AGS cells, NAT10-knockout cells and knockout cells transfected with NAT10 Δhelicase were examined by acRIP-qPCR analysis. (B) The global ac4C levels in total RNA and mRNA from AGS and BGC823 cells treated with increasing doses of Remodelin were determined by HPLC–MS/MS quantification. (C) ac4C dot blot assays of ac4C mRNA levels in AGS cells treated with Remodelin. (D) The relative ac4C levels of MDM2 mRNA in cells treated with Remodelin were examined by acRIP-qPCR analysis. (E, F) qRT–PCR (E) and Western blot (F) analyses of MDM2 expression levels in MKN45 cells expressing NAT10 shRNAs or the control. (G, H) The ac4C (G) and mRNA expression (H) levels of MDM2 in xenograft tumor tissues recovered from nude mice. (I) Luciferase reporter assays for the MDM2 promoter region. (J) Relative distribution of MDM2 mRNA in NAT10-knockout and control cells, as determined by RT–PCR. (K) MDM2 mRNA stability assessment in AGS and BGC823 cells treated with Remodelin. (L) Relative mRNA expression of TP53, CDKN1A and PUMA was measured by RT–qPCR in the indicated cells. (M) mRNA levels of CDKN1A and PUMA in AGS and BGC823 cells treated with Remodelin for 24 h. (N) Western blot analysis of p53 acetylation at K210 in the indicated cells. Error bars indicate the SD from three independent experiments. **P* < 0.05, ***P* < 0.01, ****P* < 0.001, and ns, not significant. *P* values were determined by a two-tailed t-test.


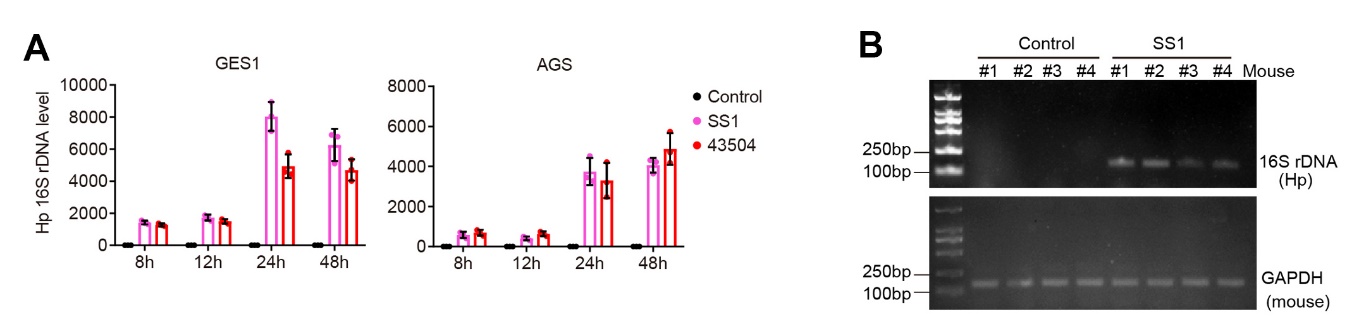


**Fig. S7. Verification of *Hp* infection by PCR analysis of *Hp* DNA.** (A) Hp-specific 16S rDNA was measured by qPCR in GES1 and AGS cells cocultured with Hp strains SS1 and 43504 for the indicated times. (B) Genomic DNA were extracted from mouse gastric tissues and Hp-specific 16S rDNA was detected by PCR.


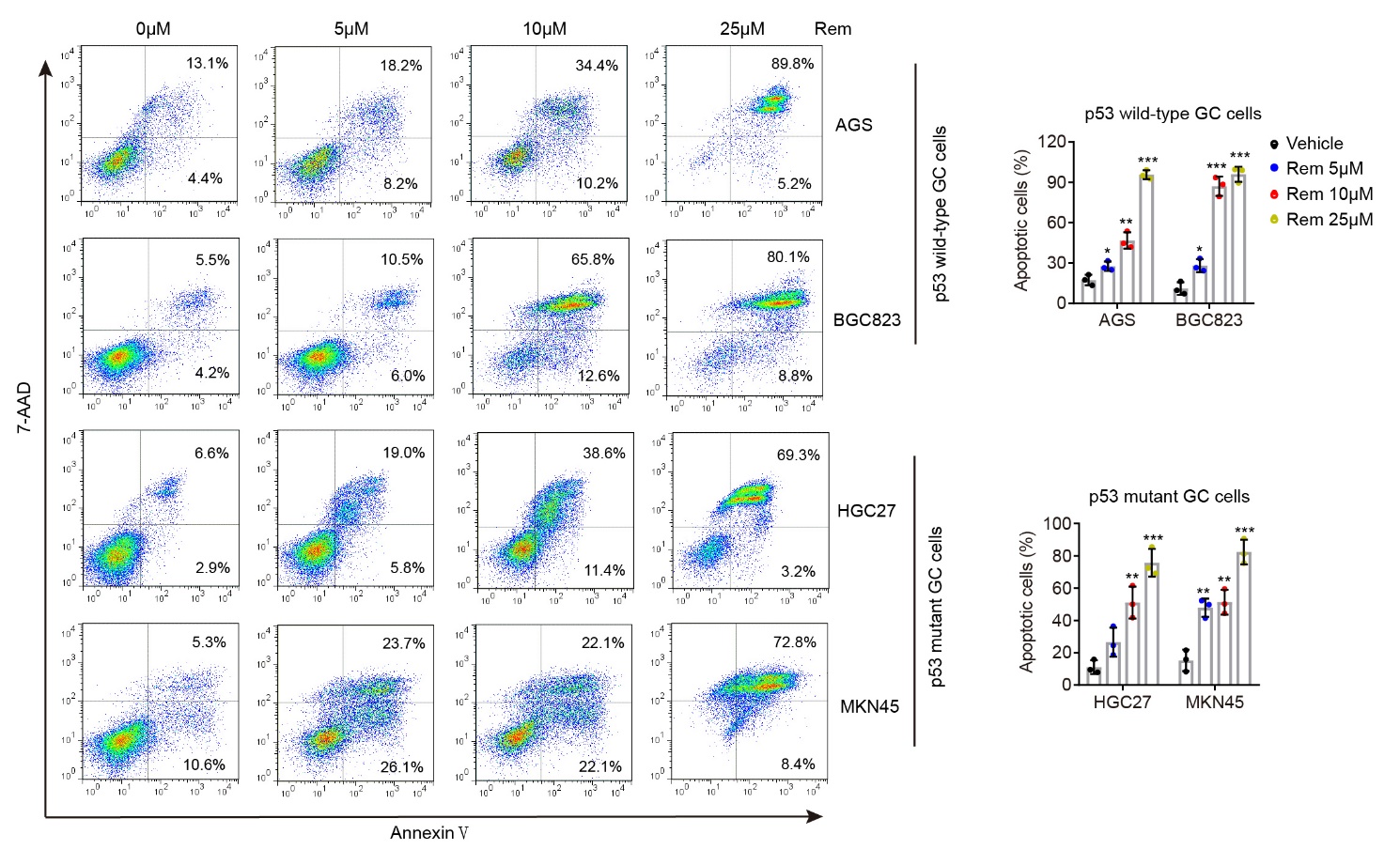


**Fig. S8. Effect of Remodelin on gastric cell apoptosis.** Flow cytometry analysis of apoptosis in GC cells following exposure to Remodelin for 48 h. Rem, Remodelin. Error bars indicate SDs from three independent experiments. **P* < 0.05, ***P* < 0.01, ****P* < 0.001. *P* values were determined by a two-tailed t-test.


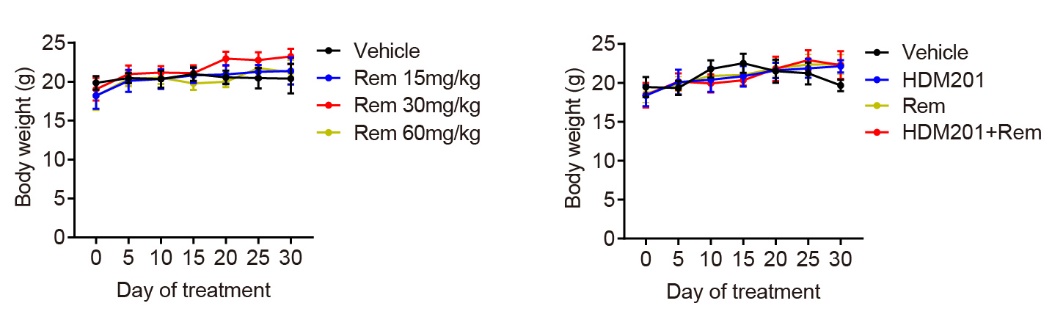


**Fig. S9. Effect of Remodelin and HDM201 on body weight of mice.** The body weight of mice bearing BGC823 subcutaneous xenografts during the course of treatment was measured every five days. Error bars, SD.


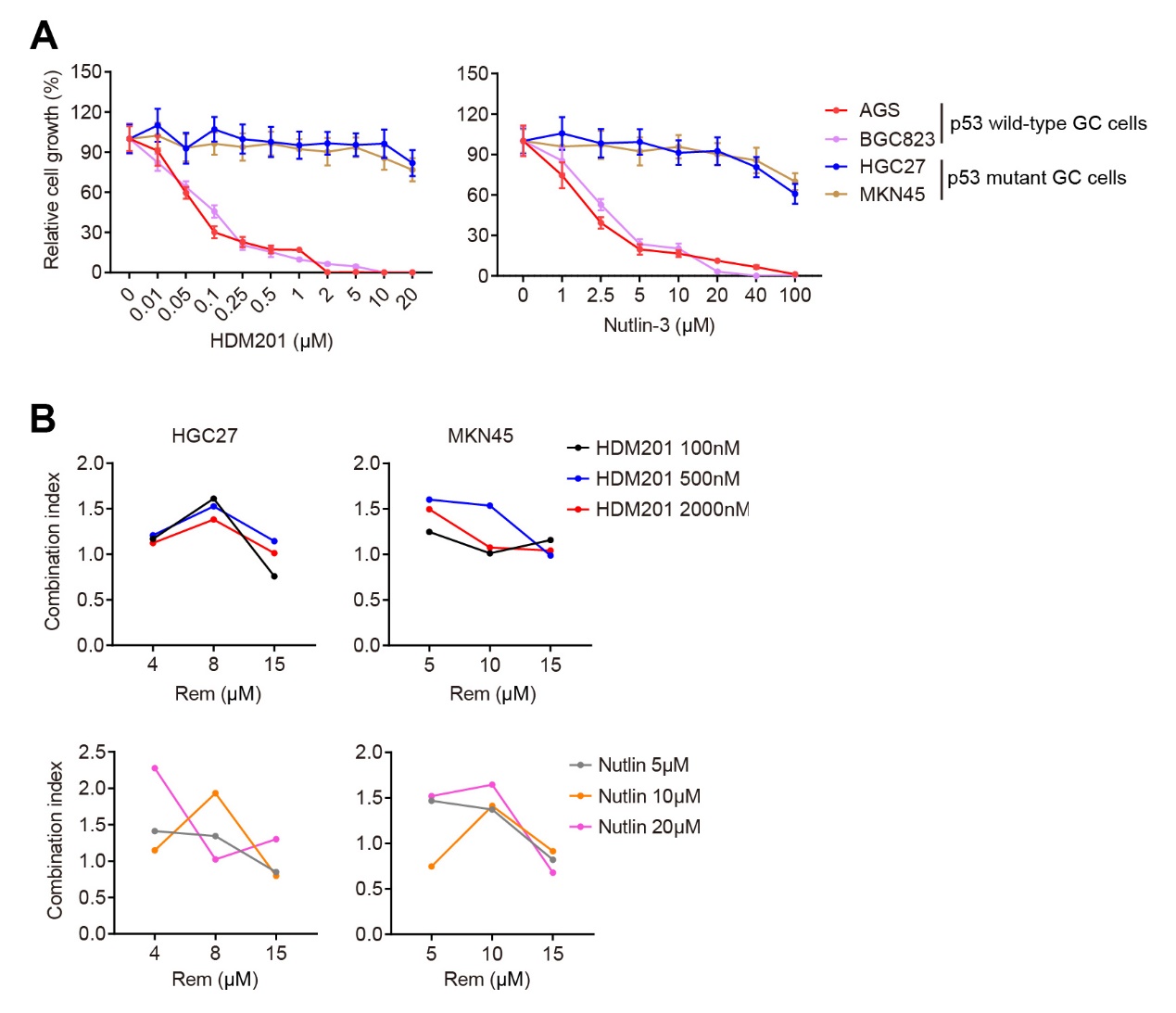


**Fig. S10. Combinatorial effects of Remodelin and MDM2 inhibitors on the proliferation of p53-mutant GC cells.** (A) Dose–response curves of gastric cancer cell lines following exposure to HDM201 or Nutlin-3 in a CCK-8 assay. (B) Synergistic analysis for Remodelin and MDM2 inhibitors (HDM201 or Nutlin-3) in the p53-mutant GC cell lines HGC27 and MKN45 based on CCK-8 assays. The CI values were determined with CompuSyn. Error bars indicate SDs from three independent experiments.

**Supplementary Table S1, S2 and S4**

**Table S1. Clinicopathological characteristics of TMA samples.**

| **Viable** | **Cases** |
| --- | --- |
| Median age (range) | 59 years (23-82) |
| Gender  Male  Female | 129 (63.9%)  73 (36.1%) |
| Histologic grade  I/II  III | 43 (21.3%)  159 (78.7%) |
| Tumor size  <5cm  ≥5cm | 140 (69.3%)  62 (30.7%) |
| Invasion depth  T1  T2  T3  T4 | 29 (14.4%)  19 (9.4%)  46 (22.8%)  108 (53.5%) |
| TNM stage  I  II  III  IV | 38 (18.8%)  43 (21.3%)  90 (44.6%)  31 (15.3%) |
| Lymph node metastasis  N0  N1  N2  N3 | 74 (36.6%)  40 (19.8%)  44 (21.8%)  44 (21.8%) |
| Distant metastasis  M0  M1 | 172 (85.1%)  30 (14.9%) |

**Table S2. Correlation between clinicopathological parameters and NAT10 levels in 202 GC tissues (χ^2^-test).**

| **Viable** | **All cases** |  | **NAT10** | | |
| --- | --- | --- | --- | --- | --- |
|  |  |  | **Low** | **High** | ***P* value** |
| Age (years)  <60  ≥60 | 104  98 |  | 47 (45.2%)  42 (42.9%) | 57 (54.8%)  56 (57.1%) | 0.738 |
| Gender  Male  Female | 129  73 |  | 54 (41.9%)  35 (47.9%) | 75 (58.1%)  38 (52.1%) | 0.403 |
| Histologic grade  I/II  III | 43  159 |  | 25 (58.1%)  64 (40.3%) | 18 (41.9%)  95 (59.7%) | **0.036** |
| Tumor size  <5cm  ≥5cm | 140  62 |  | 68 (48.6%)  21 (33.9%) | 72 (51.4%)  41 (66.1%) | 0.052 |
| Invasion depth  T1/2  T3/4 | 48  154 |  | 28 (58.3%)  61 (39.6%) | 20 (41.7%)  93 (60.4%) | **0.023** |
| TNM stage  I  II  III  IV | 38  43  90  31 |  | 23 (60.5%)  24 (55.8%)  34 (37.8%)  8 (25.8%) | 15 (39.5%)  19 (44.2%)  56 (62.6%)  23 (74.2%) | **0.007** |
| Lymph node metastasis  Absent  Present | 74  128 |  | 37 (50.0%)  52 (40.6%) | 37 (50.0%)  76 (59.4%) | 0.196 |
| Distant metastasis  Absent  Present | 172  30 |  | 81 (47.1%)  8 (26.7%) | 91 (52.9%)  22 (73.3%) | **0.038** |

**Table S4. Primers used for qRT-PCR**

| **Gene** | **Forward primer sequence (5′→3′)** | **Reverse primer sequence (5′→3′)** |
| --- | --- | --- |
| GAPDH | AATGGGCAGCCGTTAGGAAA | GCCCAATACGACCAAATCAGAG |
| ACTB | AGCACAGAGCCTCGCCTTT | ATCATCATCCATGGTGAGCTGG |
| NAT10 | TAGAAACAGTGGAAGGTGGTG | GGAGGAGATGGGCAGGA |
| MDM2 | TCTCCCTGTCTTCTCTTAGG | TCTTACTTTTCACGCTGACA |
| CDKN1A | TGTCCGTCAGAACCCATGC | AAAGTCGAAGTTCCATCGCTC |
| PUMA | GCCAGATTTGTGAGACAAGAGG | CAGGCACCTAATTGGGCTC |
| F-Luc | TCAATCTGCCCTGCTGG | GGCTTGTCGTCCCCTTC |
| U2 | ATCGCTTCTCGGCCTTT | TTCCATCTCCCTGCTCC |
